# Mulching in lowland hay meadows drives an adaptive convergence of above- and below-ground traits reducing plasticity and improving biomass: A possible tool for enhancing phytoremediation

**DOI:** 10.3389/fpls.2022.1062911

**Published:** 2022-11-24

**Authors:** Michele Dalle Fratte, Antonio Montagnoli, Simone Anelli, Stefano Armiraglio, Peter Beatrice, Alex Ceriani, Elia Lipreri, Alessio Miali, Paolo Nastasio, Bruno Enrico Leone Cerabolini

**Affiliations:** ^1^ Department of Biotechnologies and Life Sciences (DBSV), University of Insubria, Varese, Italy; ^2^ Ente Regionale per i Serivizi all’Agricoltura e alle Foreste della Lombardia (ERSAF), Milan, Italy; ^3^ Municipality of Brescia - Museum of Natural Sciences, Brescia, Italy

**Keywords:** fine-root traits, functional traits, global plant spectrum, heavy metals, leaf economics spectrum, phytoremediation, PCB, root economics spectrum

## Abstract

We aimed to understand the effect of mulching (i.e., cutting and leaving the crushed biomass to decompose in situ) on above- and below-ground plant functional traits and whether this practice may be a potential tool for enhancing the phytoremediation of lowland hay meadows. To this aim, we evaluated at the community level seven years of mulching application in a PCBs and HMs soil-polluted Site of National Interest (SIN Brescia-Caffaro) through the analysis of the floristic composition and the above- and below-ground plant traits. We found that the abandonment of agricultural activities led to a marked increase in the soil organic carbon and pH, and the over-imposed mulching additionally induced a slight increase in soil nutrients. Mulching favored the establishment of a productive plant community characterized by a more conservative-resource strategy, a higher biomass development, and lower plasticity through an adaptative convergence between above- and below-ground organs. In particular, the analysis of the root depth distribution highlighted the key role of roots living in the upper soil layer (10 cm). Mulching did not show a significant effect on plant species known to be effective in terms of PCB phytoremediation. However, the mulching application appears to be a promising tool for enhancing the root web that functions as the backbone for the proliferation of microbes devoted to organic contaminants’ degradation and selects a two-fold number of plant species known to be metal-tolerant. However, besides these potential positive effects of the mulching application, favoring species with a higher biomass development, in the long term, may lead to a biodiversity reduction and thus to potential consequences also on the diversity of native species important for the phytoremediation.

## 1 Introduction

Phytoremediation is the use of plants to remediate pollutants in contaminated soil, water, and air. It encompasses several methods for contaminant degradation, removal, or immobilization (Mackova et al., 2010). In the last 30 years, many examples described plant species accumulating and/or metabolizing organic xenobiotics, (see Mackova et al., 2010 and references therein). Despite having lots of information about the use of plants for phytoremediation purposes, much work is still necessary to forecast all aspects of its beneficial application (McCutcheon and Schnoor, 2003; Macek et al., 2004; Mackova et al., 2010), especially for recalcitrant soil contaminants such as persistent organic pollutants (POPs) and heavy metals (HMs). Regarding phytoremediation, the rhizosphere is of particular relevance since it hosts microbial communities with different metabolism that depend on the chemicals released from plant roots (McNear, 2013; Reinhold-Hurek et al., 2015). However, field testing is still needed to extend the theoretical knowledge and the practical experience learned from model plants to native plant communities.

Many hydrophobic organic compounds, including PCBs, although banned since the 1970s, are priority soil contaminants because of their toxicity and tendency to persist in soils/sediments and to escape biological degradation (Passatore et al., 2014). Therefore, PCBs phytoremediation has attracted increasing attention (Mackova et al., 2009; Mackova et al., 2010; Vergani et al., 2017; Jiang et al., 2022). At the same time, the problem of HMs’ pollution is becoming more and more severe with increasing industrialization even because, unlike organic substances, HMs are completely non-biodegradable (Mackova et al., 2010; Ali et al., 2013). Several investigations have shown that PCBs and HMs can be translocated from soil to various parts of the plants and can accumulate in higher concentrations in particular tissues than in others. Plants can uptake HMs from the soil through plant roots and translocate them to shoots (Tangahu et al., 2011; Ali et al., 2013). Conversely, plants can accumulate PCBs from the soil into the roots (Mackova et al., 2009; Terzaghi et al., 2019; Terzaghi et al., 2020) and from air into leaves even if the roots-to-leaves transfer is generally limited by the high hydrophobicity of these chemicals (Collins et al., 2006). However, the sorption of metalloids, metals, and organic compounds, is also controlled by the soil organic matter (Cornelissen et al., 2005; Branzini and Zubillaga, 2012). The application of carbon-rich charcoal-like materials such as biochar and activated carbon has been proposed, for example, as a tool for the *in-situ* stabilization of organic contaminants in soils (Beesley et al., 2011; Denyes et al., 2013). However, the relative increase in soil carbon fractions due to charcoal-like materials amendment can vary depending on the environmental conditions (Chagas et al., 2022). It is, therefore, necessary to test other methods for increasing soil organic matter based on material collected *in situ* and less affected by the multiple local factors.

Mulching has been used since the 1990s as a low-cost alternative to grazing or conventional mowing for abandoned grasslands maintenance (Mašková et al., 2009). The method consists in cutting the above-ground biomass and crushing the clippings into pieces several centimeters long that are left on the site to decompose and release a large proportion of their mineral nutrient content (Gaisler et al., 2004; Doležal et al., 2011; Metsoja et al., 2012). There have been several studies dealing with the effects of mulching on grasslands/meadows (Gaisler et al., 2019; and references therein), but only a few of these were conducted over a long period (Moog et al., 2002; Mašková et al., 2009; Römermann et al., 2009; Gaisler et al., 2013; Oelmann et al., 2017; Gaisler et al., 2019). Although mulching can represent an alternative way of increasing soil carbon content in grassland soils (Jordán et al., 2010), it has never been considered a potential tool for enhancing phytoremediation in contaminated areas. Indeed, compared to traditional management practices (i.e., grazing and/or mowing), mulching leaves above-ground biomass to decompose *in situ* and thus can be beneficial in two ways: for the phytoremediation of soil contaminants and to avoid the problem of the disposal of the contaminated biomass. However, it is not still clear whether alternative management treatments in grasslands, such as mulching, can lead to changes in floristic and functional composition.

Although mulching can have similar effects to traditional management on the floristic composition due to the removal of taller plants and prevention of succession (Pavlů et al., 2016; Gaisler et al., 2019), there is contrasting evidence about its impact on species richness (Moog et al., 2002; Mašková et al., 2009; Doležal et al., 2011; Gaisler et al., 2013; Gaisler et al., 2019). Also, its effect on the above- and below-ground plant community structure and functions represented by plant functional traits can be much different (Kahmen et al., 2002; Kahmen and Poschlod, 2008; Römermann et al., 2009; Doležal et al., 2011; Gaisler et al., 2019). Plant functional traits are key features of individual organisms related to their fitness and responses to environmental conditions (Dalle Fratte et al., 2019b) and are indeed strongly related to management measures (De Bello et al., 2010; Lavorel et al., 2011). In herbaceous plant communities, management types characterized by intermediate levels of disturbance can facilitate average levels of competitiveness, stress tolerance and ruderality, while an excessive productivity load can lead to more competitive communities (Grime, 2006a; Cerabolini et al., 2016). A slight increase in competitive and acquisitive plants with mulching treatment has been observed (Moog et al., 2002; Römermann et al., 2009; Doležal et al., 2011). Since mulching has been associated with higher soil nutrient levels (Oelmann et al., 2017) the development of more productive plant communities is reasonable, but this may negatively affect species richness (Grime, 2006a; Pierce, 2014).

Differences in land use types and management are essential for plant rooting (Ravenek et al., 2016; Tasser et al., 2021), but a complete evaluation of the effects of mulching on above- and below-ground plant traits (e.g., fine-root traits) coordination remain poorly characterized. Crucially, there is still no information concerning the effects of mulching on below-ground plant functional traits. Mowing and fertilization, which can be considered the two main factors of mulching, have been applied separately, but only the fertilization resulted in a considerable root traits shift (Leuschner et al., 2013). The increment of soil organic matter due to mulching treatment may reduce the survival of individual roots, increasing the fine-root turnover rate (Pritchard et al., 2010), resulting in a general suppression of fine-root length independently of the diameter class considered (Simiele et al., 2022). Trait-based approaches emerged in the last decades to address different ecological questions from the individual to plant-community level (Díaz et al., 2016; Bruelheide et al., 2018). However, a complete understanding of the trade-offs between above- and below-ground functional traits is difficult to reach (Carmona et al., 2021; De la Riva et al., 2021; Weigelt et al., 2021), also because root traits remain poorly investigated (Kattge et al., 2020). Specifically, Carmona et al. (2021) pointed out that the trade-offs between above-ground and fine-root traits seem decoupled. Indeed, their coordination may strongly influence plant competition, community structure, and plant-environment interactions (Tumber-Dávila et al., 2022). In particular, the more productive niche imposed by mulching may drive a functional convergence of plant traits (Grime, 2006b), and this should involve both above- and below-ground traits. Thus, it becomes crucial to investigate these two components of plant communities to get a full view of the adaptative ways of plant communities to changes in environmental features due to global changes or different management regimes.

Following the imposed abandonment in the 1990s of agricultural areas in the Site of National Interest (SIN) Brescia-Caffaro (Northern Italy) due to soil contamination by organic and inorganic pollutants, mulching has recently been introduced as a potential tool for phytoremediation of soil contaminants. In this study we focused on the effects of mulching on above- and below-ground traits of these communities, and we hypothesized that: 1) mulching favors the establishment of plant communities typical of stable hay meadows, 2) mulching drives the selection of a more productive plant community by reducing the species richness, 3) mulching led to an adaptative convergence between above- and below- ground traits at the plant community level, 4) mulching can select plant species that are metal-tolerant and/or active in PCB degradation. To test our multiple hypothesis, we analyzed the floristic-vegetation composition, above- and below-ground biomass, and community-level leaves and fine-root traits of the plant communities of the abandoned agricultural areas, and we compared their values between areas subject to mowing and mulching and areas subject to traditional mowing.

## 2 Methods

### 2.1 Study site and plot selection

We carried out this study in the agricultural areas of the Site of National Interest (SIN) Brescia-Caffaro, Northern Italy (45° 32.365’ N, 10° 11.123’ E), a site heavily contaminated by the polluted exhaust water of the factory, which was used for more than 50 years for irrigation (Di Guardo et al., 2020). The Caffaro factory was one of the larger PCB producers in Europe until 1984. In that year, the surrounding areas were found to be heavily contaminated by PCBs of more than 80 congeners, including PCB 209, the decachlorinated PCB at concentrations in the order of tens of mg kg^-1^ of total PCBs (Terzaghi et al., 2019), but also PCDDs, PCDFs, DDT and its isomers, metalloids and HMs (e.g., As up to 79 mg Kg^-1^, Hg up to 4 mg Kg^-1^, and Pb up to 447 mg Kg^-1^) exceeding the threshold concentration of contamination (Di Guardo et al., 2017). Therefore, the agricultural activity on the site has been banned for the last four decades. Since 2014, mowing and mulching have been introduced every 3 to 4 times a year. The cut biomass is then crushed into pieces 5 – 10 cm long and left in place to favor the coverage of herbaceous species capable of developing rhizosphere degradation processes while preventing the settlement of woody plant species.

**Figure 1:**
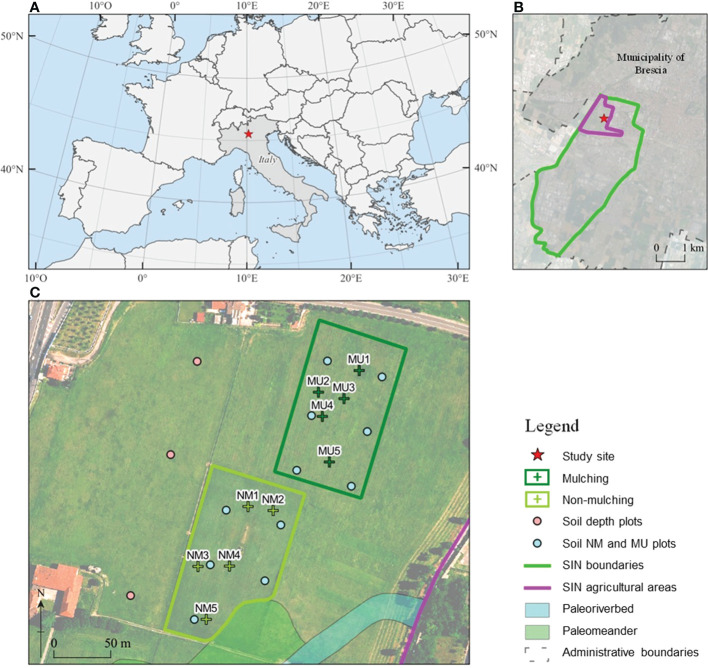
Location of the National Relevance Site (SIN) Brescia-Caffaro in Northern Italy **(A)** with a detail of the contaminated agricultural areas **(B)**, and of the control (NM; no mulching) and mulching-treated (MU) areas sampled within the study site **(C)**. Crosses indicates the location of the vegetation sampling points, and circles indicate the location of the soil sampling points: pink, soil sampled each 10 cm layer up to 100 cm depth in 2014, light blue, mixed homogeneous soil sampled up to 40 cm in 2014 and 2020.

Within the same pedologic unit, we selected two sampling areas corresponding to the control (NM; no mulching) and mulching-treated (MU) areas (**Figure 1**). These soils are deep silty loam with a poor skeleton in the topsoil, typical of coarse limestone floods (Fluventic Hapludolls; Soil Survey Staff, 2014). In 2014 they were characterized by greater cation exchange capacity (CEC) in the upper part (0 – 40 cm), pH values falling within the range 7.6 – 7.8, and almost stable up to 100 cm depth, with higher soil organic carbon (SOC), total soil nitrogen and sulfur content (respectively, N_tot_ and S_tot_) in the upper 10 cm compared to the depth 60 – 100 cm. Soil calcium and magnesium content (Ca and Mg) showed higher values in the first 30 cm depth and lower values at greater depth, while soil iron and potassium content (Fe and K) were almost stable along the depth (**Table 1** and **Supplementary Figure 1**). The maximum concentration of organic contaminants in the soils of the study site is usually within the first 40 cm of soil (Di Guardo et al., 2020).

**Table 1:** Average values (± standard deviation) of the main physico-chemical soil properties in relation to soil depth layers of the three points sampled in 2014 up to 100 cm depth (see **Figure 1**).

soil depth	sand	silt	clay	CEC	pH	SOC	N_tot_	S	Ca	Fe	Mg	K
	(%)	(%)	(%)	(meq 100g^-1^)		(g Kg^-1^)	(g Kg^-1^)	(g Kg^-1^)	(g Kg^-1^)	(g Kg^-1^)	(g Kg^-1^)	(g Kg^-1^)
**0-10**	57.00 ± 10.34	35.60 ± 9.70	7.38 ± 0.99	24.63 ± 1.94	7.76 ± 0.19	20.27 ± 5.98	2.18 ± 0.32	0.38 ± 0.02	32.07 ± 7.20	28.2 ± 1.5	22.93 ± 3.04	11.40 ± 0.53
**10-20**	55.34 ± 9.91	35.00 ± 8.65	9.63 ± 1.44	23.63 ± 1.19	7.59 ± 0.08	13.73 ± 8.95	1.53 ± 0.17	0.28 ± 0.04	28.73 ± 5.25	25.87 ± 4.16	21.43 ± 4.97	10.16 ± 1.67
**20-30**	52.17 ± 12.76	38.34 ± 12.95	9.52 ± 0.86	22.67 ± 1.17	7.80 ± 0.26	14.67 ± 7.68	1.42 ± 0.30	0.29 ± 0.01	31.63 ± 5.76	28.87 ± 2.87	22.77 ± 3.18	9.78 ± 1.67
**30-40**	52.60 ± 7.49	36.43 ± 6.26	10.96 ± 1.28	22.60 ± 1.39	7.76 ± 0.09	15.00 ± 6.01	1.08 ± 0.33	0.26 ± 0.04	34.53 ± 6.32	28.4 ± 2.49	26.17 ± 4.76	10.97 ± 3.02
**40-60**	46.60 ± 3.24	41.87 ± 4.27	11.50 ± 1.01	21.43 ± 1.36	7.79 ± 0.07	10.63 ± 5.19	0.68 ± 0.17	0.20 ± 0.02	35.50 ± 8.25	27.97 ± 1.42	26.97 ± 5.56	10.94 ± 1.94
**60-80**	48.52 ± 7.02	40.03 ± 5.61	11.43 ± 1.45	18.70 ± 1.31	7.86 ± 0.03	10.73 ± 7.71	0.42 ± 0.30	0.17 ± 0.03	42.77 ± 2.20	27 ± 2.17	28.33 ± 3.71	10.55 ± 1.36
**80-100**	43.56 ± 6.78	44.93 ± 6.66	11.47 ± 0.76	16.33 ± 1.10	7.96 ± 0.15	7.08 ± 6.13	0.25 ± 0.01	0.14 ± 0.03	49.83 ± 1.46	25.13 ± 2.68	29.80 ± 4.39	9.54 ± 0.55
**ALL**	50.83 ± 8.60	38.89 ± 7.67	10.27 ± 1.74	21.43 ± 3.01	7.79 ± 0.16	13.16 ± 7.02	1.08 ± 0.68	0.24 ± 0.08	36.44 ± 8.46	27.35 ± 2.56	25.49 ± 4.69	10.48 ± 1.58

CEC, cation exchange capacity; SOC, organic carbon; Ntot, total nitrogen; Stot, total sulfur; Ca, calcium; Fe, iron; Mg, magnesium; K, potassium.

Both NM and MU areas had an extension of about 1.1 ha (**Figure 1**). The NM area was characterized by the mowing practices carried out regularly over the years, which implies the removal of cut material. Before mulching treatment (2014), the study site was homogeneously characterized by synanthropic and ruderal vegetation. This vegetation type was related to the cultivation abandonment that started in the 1980s. The SIN agricultural area has a plain surface with a negligible elevation difference, less than 70 cm for the study site (**Figure 1**). Indeed, we focused on a single field of the study site to avoid any potential bias due to the legacy of different past agricultural activities. Moreover, the spatial distribution of NM and MU areas has been based on the ongoing management activities established by the responsible organization (ERSAF – Ente Regionale per i servizi all’Agricoltura e alle Foreste, Lombardia).

### 2.2 Sampling and data collection

#### 2.2.1 Experimental design and floristic-vegetation analysis

During the 2021 within each area (NM and MU), five points were randomly selected for vegetation analysis, using a minimum distance of 20 m from each other and an internal buffer of 10 m to avoid any disturbances due to edge effects. Each point corresponds to the center of a 4 x 4 m squared plot for a total of ten floristic-vegetation relevés (five in the NM and five in the MU areas). The coverage of all vascular plant species was determined by visual estimation and recorded directly on a percentage scale. The plant species evaluated for their phytoremediation capability of PCB-contaminated soils (Vergani et al., 2017) and known as metal-tolerant (Landolt et al., 2010) were listed for each relevé.

The nomenclature used for plant species is based on the most recent checklist of the native and alien vascular plants of Italy (Bartolucci et al., 2018; Galasso et al., 2018). Within each plot, a 2 x 2 m core area was selected and divided into 16 subplots of 50 x 50 cm to measure the biomass. Two of these subplots were randomly selected for a total of 20 biomass samples (ten in the NM and ten in the MU areas). The floristic-vegetation relevés and the biomass sampling were carried out on the same days (24 and 25 June 2021), coinciding with the estimated peak biomass. In the center of each subplot, we then sampled 10 cm soil cores (diameter and height of 4 and 10 cm, respectively), reaching a depth of 40 cm using a motor-driven core drill. We selected a depth of 10 cm interval because this is the standard unit for sampling root biomass considering depth increments (e.g., Fort et al., 2013; Freschet et al., 2021a; Baronti et al., 2022).

#### 2.2.2 Above-ground traits analysis

For each species, leaf functional traits representative of the plant size and economics (i.e., leaf area, LA; leaf dry matter content, LDMC; specific leaf area, SLA; leaf nitrogen content, LNC) were obtained from the authors’ datasets (FIFTH and LIFTH, see Cerabolini et al., 2010; Dalle Fratte et al., 2021) accessible through the TRY database (Kattge et al., 2020, https://www.trydb.org: see datasets n. 227, 228, 229, 371, 372 and the forthcoming 467). Above-ground standing crop and litter (Al-Mufti et al., 1977) were sampled in the whole 50 x 50 cm subplot using an electric lawn mower and oven dried (70°C for 24 h) to obtain the above-ground dry weight (AGDW).

#### 2.2.3 Below-ground traits analysis

The soil cores were immediately stowed in a portable refrigerator and then stored in the laboratory at 4°C until their analysis. Each sample of soil cores was placed in a nylon bag (400 μm mesh) closed at one end with a zip tie; each nylon bag was inserted into the washing machine drum and automatically washed with cold water to let the soil sieve out so that only roots and stones remained within the nylon bag (adapted from Benjamin and Nielsen, 2004). Within each washed nylon bag, all the fine-root material was separated from the rest (organic and mineral fraction of the soil and stone materials) using tweezers and a stereomicroscope.

The root samples were immersed in water in order to avoid drying and consequent shrinkage and scanned at a resolution of 800 dpi with a calibrated scanner coupled with a transparency unit (Epson Expression 10,000 XL). We estimated the below-ground dry weight (BGDW) of each 10 cm soil layer by drying it in an oven at 70°C for 24 h. Finally, the scanned images were analyzed with WinRhizo Pro V. 2007d software (Regent Instruments Inc., Quebec, Canada) to obtain morphological data such as root length (RL) and mean root diameter (MRD), as well as RL in each diameter class of 0.1 mm interval.

#### 2.2.4 Soil chemical analysis

In 2014 and 2020 we also analyzed soil characteristics such as pH, SOC and N_tot_ in the upper 40 cm of soil in 11 points distributed within each sampling area (five in the NM and six in the MU areas) (**Figure 1**). We sampled soil cores up to a depth of 40 cm; the entire length was mixed and used for the chemical analysis.

### 2.3 Data analysis

To determine the mulching effect on the floristic composition of plant communities we calculated the indicator species for each of the two groups of relevés (NM vs. MU areas) using the “multipatt” function of the “indicspecies” R package (De Cáceres and Legendre, 2009); we applied the correlation index based on abundance data (r.g) which is more sensitive to the local ecological context.

For each soil depth layer (0 - 10, 10 - 20, 20 - 30, and 30 - 40 cm) we calculated the following root traits: below-ground dry weight (BGDW), mean root diameter (MRD), root length (RL), specific root length (SRL), and fine-root percentage (FRP). The FRP was calculated as the percentage of RL with a diameter < 0.1 mm (Fort et al., 2013); SRL was calculated by dividing the RL by BGDW (Ostonen et al., 2007; Pérez-Harguindeguy et al., 2013; Montagnoli et al., 2019). For each root trait and class of soil depth, we first calculated the average value between the two subplots, and we then summarized the same traits at the plot-level (0 - 40 cm) as following: RL and BGDW were summed among all soil depth layers, while SRL, MRD and FRP were calculated as the average value among all soil depth layers. The same procedure was also used to calculate the total RL in each class of diameter.

For each relevé we calculated the community weighted mean (CWM) of leaf traits, using as weight the estimated percentage coverage of each species. To estimate the AGDW of each plot we also calculated the average value between the two subplots. The total biomass for each plot was calculated as the sum of the AGDW and BGDW of each plot. In each of the two groups of relevés (NM vs. MU areas) we then analyzed the relations between biodiversity (i.e., species richness) and productivity (i.e., total biomass) fitting a generalized linear model (GLM) by means of the function “glm” of the “stats” base R package. We compared the community-level traits (both above- and below-ground) between NM and MU areas by means of the Wilcoxon test.

A redundancy analysis (RDA) was performed to determine the relations between community-level explanatory variables (i.e., CWM of leaf traits and community-level root traits, above- and below-ground biomass) that resulted to have a significant effect on the previous analysis and floristic composition of plant communities. For this aim, we used the function “rda” of the “vegan” R package (Oksanen et al., 2022). Before RDA, data were scaled to unit variance. We tested the significance of each constrained axis independently through a permutation test based on 999 randomizations of the rows of the environmental matrix to preserve the correlation between environmental variables (De Bello et al., 2021).

We then compared the differences of soil physico-chemical properties along depth (3 points in 2014) and between NM and MU (12 points in 2014 vs. 2020) by means of the Dunn’s test multiple comparisons using the function “dunn.test” of the package “dunn.test” (Dinno, 2017). The same analysis was also used to compare root traits in relation to soil depth.

All the analyses were done using the R software (R Core Team, 2021).

## 3 Results

### 3.1 Floristic composition and vegetation structure

Considering all the relevés we found a total of 38 vascular plant species, 27 in the NM area and 33 in the MU area (**Table 2**). The indicator plant species of the MU area were *Arrhenatherum elatius*, *Sorghum halepense*, *Galium mollugo*, and *Convolvulus sepium*, while indicator species in the control area were *Daucus carota*, *Trifolium pratense*, and *Achillea roseoalba* (**Table 2**). The analyzed relevés did not highlight native plant species known for their significance in conservation or exotic plant species belonging to the regional blacklist of invasive alien species (LR 10/2008 and subsequent updates).

**Table 2:** Synoptic table of the frequency and average coverage of the species in the 10 floristic-vegetation relevés carried out in the control (no-mulching; NM) and mulching-treated (MU) areas.

		Indicator species	Frequency (%)		Coverage (%)		Phyto-rhizoremediation	Metal tolerant
Species name			NM	MU	NM	MU		
Daucus carota L.	Dau_car	** (NM)	100	100	52	6	p	m
Trifolium pratense L. subsp. pratense	Tri_pra	* (NM)	100	80	20	4	p	m
Achillea roseoalba Ehrend.	Ach_ros	* (NM)	100	60	20	3		
Avena barbata Pott ex Link	Ave_bar		80	.	1	.		
Myosotis arvensis (L.) Hill subsp. arvensis	Myo_arv		40	.	1	.		m
Picris hieracioides L. subsp. hieracioides	Pic_hie		20	.	1	.		
Trifolium repens L.	Tri_rep		20	.	1	.		m
Trifolium campestre Schreb.	Tri_cam		20	.	1	.		m
Arrhenatherum elatius (L.) P.Beauv. ex J.Presl & C.Presl subsp. elatius	Arr_ela	* (MU)	100	100	44	73		m
Sorghum halepense (L.) Pers.	Sor_hal	** (MU)	100	100	10	40		
Galium mollugo L.	Gal_mol	** (MU)	100	100	2	10		m
Convolvulus sepium L.	Con_sep	** (MU)	.	100	.	10		m
Medicago lupulina L.	Med_lup		.	60	.	1	p	m
Vicia sativa L.	Vic_sat		.	40	.	1		
Verbena officinalis L.	Ver_off		.	40	.	2		
Rumex crispus L.	Rum_cri		.	40	.	1	p	m
Rumex acetosa L. subsp. acetosa	Rum_ace		.	40	.	1		m
Veronica persica Poir.	Ver_per		.	40	.	1		m
Clematis vitalba L.	Cle_vit		.	40	.	1		m
Hypericum perfoliatum L.	Hyp_per		.	20	.	2		
Taraxacum F.H.Wigg. sect. Taraxacum	Tar_off		.	20	.	1		m
Lathyrus sp.	Lat_sp.		.	20	.	1		
Erigeron annuus (L.) Desf.	Eri_ann		100	80	6	2		m
Cerastium brachypetalum Desp. ex Pers. subsp. brachypetalum	Cer_bra		100	80	1	1		m
Lotus corniculatus L. subsp. corniculatus	Lot_cor		80	100	13	8		m
Plantago lanceolata L.	Pla_lan		100	60	10	2		m
Lolium perenne L.	Lol_per		100	40	13	4	p	m
Dactylis glomerata L. subsp. glomerata	Dac_glo		80	20	2	1		m
Convolvulus arvensis L.	Con_arv		80	20	9	5		m
Cirsium arvense (L.) Scop.	Cir_arv		40	100	4	6	p	m
Medicago sativa L.	Med_sat		40	40	8	2	p	
Salvia pratensis L. subsp. pratensis	Sal_pra		80	60	19	4		
Clinopodium vulgare L. subsp. vulgare	Cli_vul		40	20	1	3		
Holcus lanatus L. subsp. lanatus	Hol_lan		60	40	6	1		m
Crepis vesicaria L.	Cre_ves		40	20	1	1		
Carex divulsa Stokes	Car_div		20	40	1	2		
Lysimachia arvensis (L.) U.Manns & Anderb. subsp. arvensis	Lys_arv		20	40	1	1		m
Bellis perennis L.	Bel_per		20	20	1	1		m

The last two columns indicate the species of interest for the phytoremediation of PCB contaminated soils (Vergani et al., 2017) and metal-tolerant species (M indicator, Landolt et al., 2010). *,p-value < 0.05, **,p-value < 0.01.

We found 25 metal-tolerant species and seven species of particular interest for the phyto-rhizoremediation of PCB-contaminated soils (**Table 2**). Among the metal-tolerant species, three were exclusive (i.e., present in a single area only) of the NM area, and seven of the MU area. Indicator plant species recognized as metal-tolerant for the NM area were *Daucus carota* and *Trifolium pratense*, and for the MU area were *Arrhenatherum elatius*, *Convolvulus sepium*, and *Galium mollugo* (**Table 2**). For both NM and MU areas, the remaining plant species had low frequency (< 2%) or scarce coverage (< 5%) and were not exclusive to one specific area (e.g., *Lolium perenne*, *Holcus lanatus*; **Table 2**). The species of interest for phyto-rhizoremediation were two in the NM area (*Daucus carota* and *Trifolium pratense*) and two in the MU area (*Cirsium arvense* and *Medicago lupulina*). The three other species of interest for phyto-rhizoremediation (*Lolium perenne*, *Medicago sativa*, and *Rumex crispus*) had high frequency (> 40%) but no significant coverage (< 15%) in both NM and MU areas (**Table 2**).

### 3.2 Above- and below-ground traits

**Figure 2:**
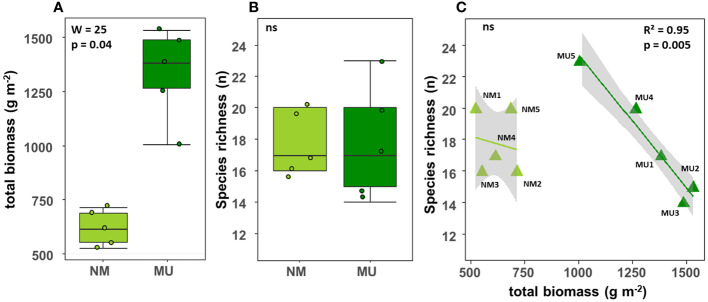
Comparison of the total biomass **(A)** and species richness **(B)** between control (NM; no mulching) and mulching-treated (MU) areas, and relationship between species richness and total biomass **(C)** in the 10 sampling plots. The lines in figure C indicate the best fitting and its 95% confidence interval obtained through a generalized linear model (GLM). The boxplot indicates the median (line in the middle of the boxes), the interquartile range (boxes) and 1.5 times the interquartile range (whiskers). Results of the Wilcoxon test **(A, B)** or GLM model **(C)** are reported in each subfigure; the R^2^ value is the pseudo-R^2^ of the GLM model; ns, not significant (p > 0.05).

The total biomass (above- plus below-ground) was significantly (p < 0.05) higher in the MU area (mean 619.3 ± SD 82.5 g m^-2^) than in the NM area (mean 1335.3 ± SD 210.6 g m^-2^) (**Figure 2A**). On the contrary, there was no significant difference in species richness between the NM (mean 17.8 ± SD 2.0 No.) and MU area (mean 17.8 ± SD 3.7 No.) (**Figure 2B**). For the MU area, plant species richness exhibited a significant (p < 0.01) linear decrease with the increase of total biomass (**Figure 2C**). On the contrary, in the NM area, we did not find significant relationship between species richness and biomass (**Figure 2C**). The species richness was higher at intermediate values of the total biomass (plot MU5), and it was lower in correspondence of both the lowest and the highest total biomass values (**Figure 2C**).

**Figure 3:**
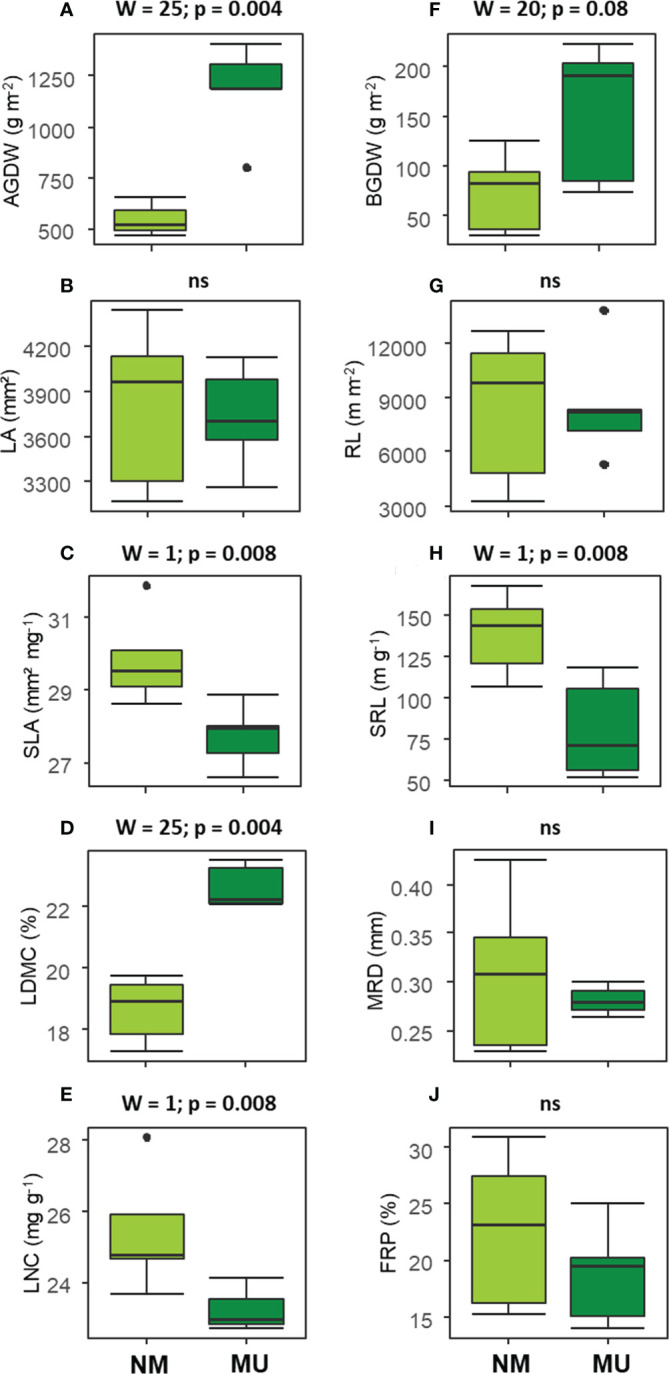
Comparison of the community-level above- **(A–E)** and below-ground traits **(F–J)** between control (NM; no mulching) and mulching (MU) in the 10 sampling plots. The boxplot indicates the median (line in the middle of the boxes), the interquartile range (boxes), 1.5 times the interquartile range (whiskers) and outliers (circle). Results of the Wilcoxon test are reported in each subfigure; ns, not significant (p > 0.1). AGDW and BGDW, above- and below- ground dry weight, FRP, fine-root percentage, LA, community weighted mean (CWM) of leaf area, LDMC, CWM of leaf dry matter content, LNC, CWM of leaf nitrogen content, MRD, mean root diameter, SLA, CWM of specific leaf area, SRL, specific root length, RL, root length.

The analysis of above-ground traits showed significantly (p < 0.01) higher values of AGDW and LDMC in the MU area (**Figures 3A, D**). On the contrary, SLA and LNC were found significantly (p < 0.01) lower in the MU than in the NM areas (**Figures 3C, E**). Finally, LA did not show any significant difference (**Figure 3B**).

Concerning the below-ground traits, BGDW (p < 0.1) and SRL (p < 0.01) showed a significant increase and decrease, respectively, in relation to mulching treatment (**Figures 3F, H**). All other traits did not show significant differences (**Figures 3G, I, J**).

**Figure 4:**
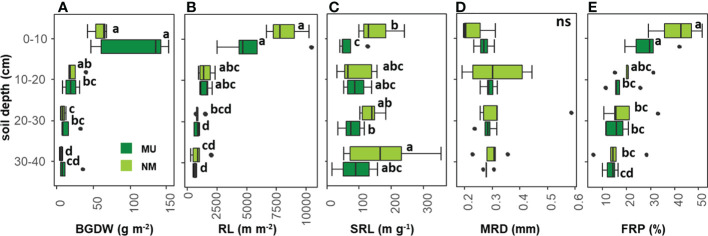
Comparison of community-level below-ground traits between control (NM; no mulching) and mulching-treated (MU) areas for each soil depth layer. The boxplot indicates the median (line in the middle of the boxes), the interquartile range (boxes), 1.5 times the interquartile range (whiskers) and outliers (circle). Small letters indicate the results of the Dunn test *post-hoc* comparisons; ns, not significant (p > 0.05). BGDW, below-ground dry weight **(A)**; RL, root length **(B)**; SRL, specific root length **(C)**; MRD, mean root diameter **(D)**; FRP, fine-root percentage **(E)**.

According to the rooting depth distribution, BGDW was not different between NM and MU areas for all the soil depth layers analyzed (**Figure 4A**). In the NM area, BGDW did not differ between the first (0 – 10 cm) and the second (10 – 20 cm) soil layer while significantly (p < 0.05) decreasing at greater soil depth (20 – 30 and 30 – 40 cm). For the MU area, BGDW in the upper soil layer (0 – 10 cm) was significantly (p < 0.05) higher than in the lower soil depth layer (10 - 20 cm). At further depth (20 – 30 cm and 30 – 40 cm) BGDW in the MU area did not significantly differ (**Figure 4A**).

RL was not different between NM and MU areas for all the soil depth layers analyzed (**Figure 4B**). In the NM area, RL in the first soil layer was significantly (p < 0.05) higher only compared to the third and fourth depth layers (20 – 30 cm and 30 – 40 cm, respectively), but RL did not differ among different soil layers, except the first one. The same pattern was also observable in the MU area, even if the third and fourth layers showed significantly (p < 0.05) lower values of RL compared to the second layer (10 – 20 cm, **Figure 4B**).

In the NM area, SRL was significantly (p < 0.05) higher than in the MU area only for the upper soil layer (0 – 10 cm) while at further depth there was no difference (**Figure 4C**). In the NM area, SRL did not differ between the first, second and third layers, while it was significantly (p < 0.05) higher at the lowest soil depth (30 – 40 cm). In the MU area, SRL in the upper soil layer was significantly (p < 0.05) lower only compared to the third layer, but there was no difference comparing the other soil depth layers (**Figure 4C**).

MRD did not show significant differences between NM and MU areas for all the soil depth layers, and among the different soil depth layers in both NM and MU areas (**Figure 4D**).

FRP was not different between NM and MU areas for all the soil depth layers (**Figure 4E**). In the NM area, FRP in the first soil layer was significantly (p < 0.05) higher only compared to the third and fourth depth layers (20 – 30 cm and 30 – 40 cm, respectively), but it did not differ among different soil layers, except the first one. In the MU area, FRP in the first soil layer was significantly (p < 0.05) higher compared to all the other soil layers, but it did not differ among the other soil layers (**Figure 4E**).

**Figure 5:**
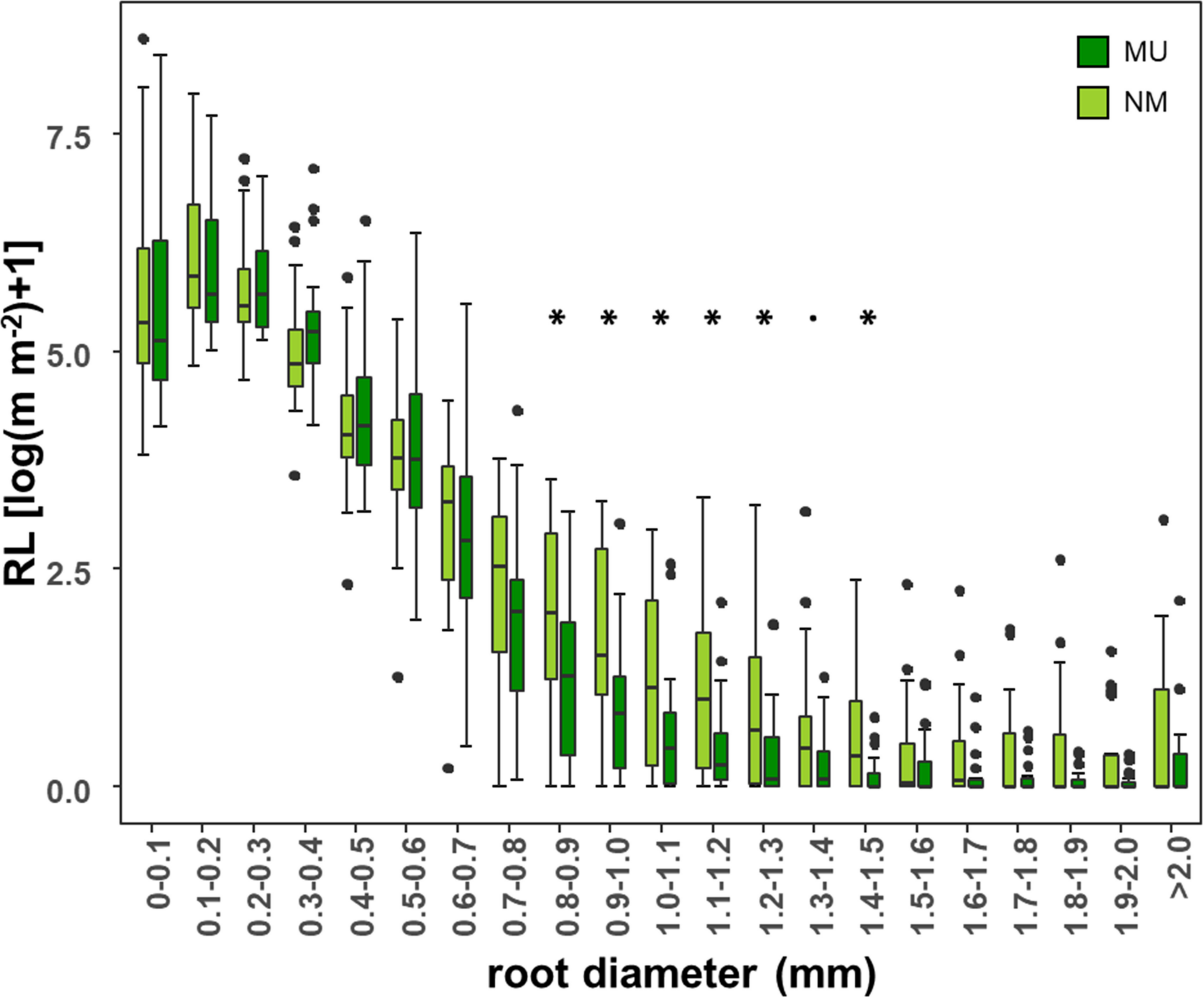
Comparison of root length (RL) within each class of root diameter at the community-level between control (NM; no mulching) and mulching-treated (MU) areas in the 10 sampled plots. RL is plotted as logarithmic only for facilitating the visualization of smaller values. The boxplot indicates the median (line in the middle of the boxes), the interquartile range (boxes), 1.5 times the interquartile range (whiskers) and outliers (circle). Results of the Wilcoxon test are reported only if significant at p < 0.05 (asterisk) or p < 0.1 (point).

According to different root diameter classes, both NM and MU areas showed higher values of RL within the very-fine fraction (d < 0.4 mm). The NM area had significantly (p < 0.05, except p < 0.1 for the class 1.3 – 1.4 mm) higher values of RL compared to the MU one for the roots falling within the diameter range of 0.8 – 1.5 mm (**Figure 5**).

### 3.3 Soil chemical properties

**Figure 6:**
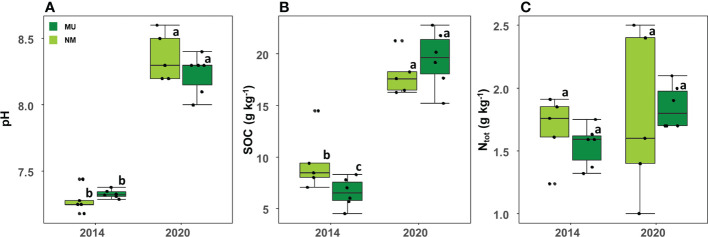
Comparison of soil pH **(A)**, organic carbon (SOC; **B**) and total nitrogen content (N_tot_; **C**) between control (NM; no mulching) and mulching-treated (MU) areas in the 11 soil plots sampled in the years 2014 and 2020. Small letters indicate the results of the Dunn test *post-hoc* comparisons.

Soil pH was significantly (p < 0.01) higher in 2020 (mean 8.3 ± SD 0.2) than in 2014 (mean 7.3 ± SD 0.1) independently of the treatment, but it did not differ between the NM and MU areas in the two sampled time-points (**Figure 6A**).

SOC also was significantly (p < 0.01) higher in 2020 (mean 18.8 ± SD 2.5 g Kg^-1^) compared to 2014 (mean 7.9 ± SD 2.6 g Kg^-1^) independently of the treatment (**Figure 6B**). In 2014 SOC was significantly higher in the NM area than in the MU one, while in 2020 SOC was slightly higher in the MU area, even if without statistical significance (**Figure 6B**).

No differences were detected between 2014 and 2020 for N_tot_ independently of the treatment (**Figure 6C**). Although without significant difference, the MU area showed a slight increase of N_tot_ from 2014 to 2020, and a simultaneous slight decrease in the NM area (**Figure 6C**).

### 3.4 Redundancy analysis (RDA)

**Figure 7:**
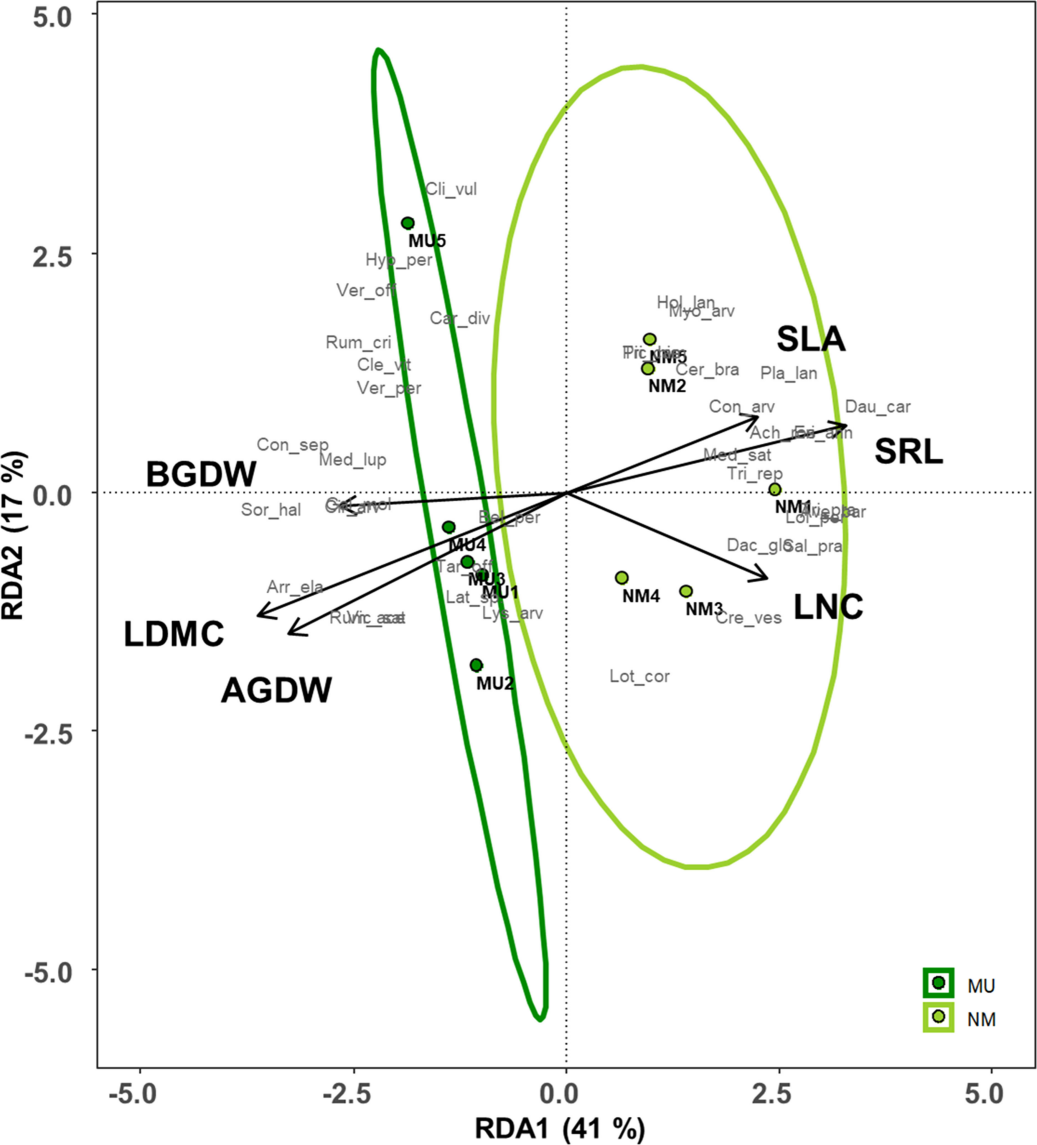
Redundancy analysis (RDA) ordination of the plant community composition comparing control (NM; no mulching) and mulching-treated (MU) areas in relation to the community-level plant functional traits that were significantly affected by the mulching treatment (see **Figures 3**). The circles represent the 95% confidence interval. Legend: AGDW and BGDW, above- and below- ground dry weight, LDMC, community weighted mean (CWM) of leaf dry matter content, LNC, CWM of leaf nitrogen content, SLA, CWM of specific leaf area, SRL, specific root length. Supporting data are reported in **Supplementary Table 1**.

Plant traits significantly affected by the mulching treatment were used for the RDA (see **Figure 4**). These traits explained 72% of the total variance of the vegetation dataset (i.e., constrained variance), and the first two axes represented 58% of this variation (**Figure 7** and **Supplementary Table 1**). Moreover, only the first axis showed a significant effect (F = 3.3, p < 0.05).

All the MU relevés were displayed on the left side of the ordination diagram and were positively correlated with both above- and below-ground biomass (AGDW and BGDW) and LDMC (**Figure 7**). On the opposite, the NM relevés were displayed on the right side of the ordination diagram correlating with SLA, LNC, and SRL (**Figure 7**). Finally, the functional space of the NM relevés was wider than MU ones.

## 4 Discussion

Our results highlighted that the plant communities of the abandoned agricultural fields of the Site of National Interest (SIN) Brescia-Caffaro are today typical of hay meadows of the class *Molinio-Arrhenatheretea* in both NM and MU areas. This was expectable due to the natural succession of spontaneous vegetation after the abandonment of agricultural activities (Prévosto et al., 2011; Sojneková and Chytrý, 2015). In particular, we found evidence that seven years of mulching favors the establishment of vegetation *facies* greatly dominated by *Arrhenatherum elatius*, thus indicating a stable vegetation *facies* of hay meadows. Moreover, our results confirmed that mulching improves the total biomass of the analyzed plant communities, both above- and below-ground (**Figures 2**, **3**), representing more productive and competitive vegetation. In a relatively fertile *A. elatius* community, we found a significant deviation in the floristic composition of the treated area (i.e., mulching - MU) compared to the control one (i.e., no mulching - NM). Comparable observations linked the floristic changes to the presence of eutrophic soils (Moog et al., 2002), which support the abundance of competitive strategist species (Grime, 2006a). On the contrary, in the control area, the vegetation evolved toward a hay meadow dominated by *Daucus carota*, representing a more ruderal vegetation *facies* (**Table 2**). In the MU area, although species richness was not significantly affected, we observed a significant linear decrease with the increase of the biomass (**Figure 2C**), thus resembling the more productive extreme of the humped-back curve (Grime, 1973; Pierce, 2014; Cerabolini et al., 2016). Such evidence suggests that long-term mulching application could lead to a reduction of biodiversity. Other long-term mulching experiments reported similar findings for semi-natural meadows dominated by *Festuca rubra* or *A. elatius* (Gaisler et al., 2013; Gaisler et al., 2019; respectively). Therefore, we may assert that mulching affects the floristic composition of plant communities favoring potentially dominant species at the expense of subordinate species. Altogether, these findings supported our first two hypothesis.

Since 2014, we observed a simultaneous increase in soil pH and SOC in both NM and MU areas (**Figure 6**). This finding might be due to the increment of soil nutrients and the release of disturbance due to the abandonment of agricultural activity (Novara et al., 2014; Nadal-Romero et al., 2021). Despite more fertile soils (i.e., higher carbon content) are usually associated with lower pH values in temperate grassland ecosystems (Kidd et al., 2017), the release of soil disturbance may have enhanced the soil biota community and related bioturbation (Kurganova et al., 2019). An increase in soil pH has been, for example, attributed to top-soil bioturbation (Dostál et al., 2005; Jílková, 2008; Desie et al., 2020), which in turn facilitates the phytoavailability of metals (Leveque et al., 2014) as well as other organic contaminants, such as PCBs that are mainly transported with solid material (Cousins et al., 1999).

When both SOC and N_tot_ parameters were compared between NM and MU areas, we found that in 2014 the MU area had slightly lower values than the NM one, while an opposite trend was observed seven years later (2020), with higher values in the MU area (**Figure 6**). Although this trend was observable, differences among data were not statistically significant, likely due to the mid-term study period (2014-2020), which is still not enough to denote appreciable differences. Indeed, it has been demonstrated that long-term mulching can significantly increase the soil organic matter and available nutrients due to the degradation of mulching materials (Jordán et al., 2010; Doležal et al., 2011). Moreover, in upland meadows, a higher nutrient content has also been associated with additional nitrogen input through N_2_ fixation of legumes favored by mulching, such as *T. repens* (Gaisler et al., 2004; Pavlů et al., 2016). In our study, legume species were equally spread in both NM and MU areas (**Table 2**), suggesting that mulching effects on legume coverage might be related to elevation where meadows are located. However, all the *Trifolium* species were associated with the NM area (**Table 2**), which in turn was characterized by low graminoids abundance, supporting the nitrogen-based competitive trade-off between grass and clover (Schwinning and Parsons, 1996).

Moreover, the accumulation of litter characterized the mulching areas with a continuous soil cover over the season. In turn, the soil cover may reduce the water runoff, soil loss, and POP mobilization and be partly responsible for different species composition compared to the control area (Moog et al., 2002; Jordán et al., 2010). However, for the same vegetation type and climatic conditions, the decomposition time of the organic matter is four weeks circa (Moog et al., 2002 and references therein), indicating a lack of effects related to the litter accumulation. Finally, mulching reduces water evaporation and increases infiltration (Gupta and Gupta, 1986), generally enhancing soil water conservation (Jun et al., 2014; Li et al., 2020). These new ecological conditions characterizing the MU area, in our study may be related to optimal condition for the spread of competitive and invasive species such as *Sorghum halepense* (**Table 2**; Dalle Fratte et al., 2019a).

Data of above- and below- ground traits showed a coordinated arrangement in the bi-dimensional space of the RDA along the first axis, which represents the economics spectrum (Wright et al., 2004; Reich, 2014), and the existence of trait syndromes at the plant community level (Zanzottera et al., 2020), which was independent of the treatment (**Figure 7** and **Supplementary Table 1**). These findings showed an adaptative convergence between leaf and root economics spectrum and, thus, a coordination of above- and below-ground organs. In detail, mulching treatment seemed to select species with a higher biomass development both above- and below-ground, supporting our third hypothesis. On the contrary, plant community of control area had a higher SLA and SRL. Mulching thus selected a conservative and more productive plant community that might be related to a higher biomass development while, the control area was characterized by acquisitive and less productive plant community, which adopted a coordinated above- and below- ground plastic strategy. Finally, we might assert that in our lowland hay meadows, SRL is the below-ground equivalent to SLA since their role is plastically enhance resource acquisition (Eissenstat and Yanai, 1997). Indeed, other authors found similar results at the species-level across different biomes (Freschet et al., 2010; Fort et al., 2013; De la Riva et al., 2019; Shen et al., 2019; Baronti et al., 2022).

Concerning the leaf economics spectrum, results reported in the literature are controversial. In oligotrophic mountain meadows, Doležal et al. (2011) found that mulching promotes species characterized by acquisitive strategies. On the contrary, in line with our findings, Oomes et al. (1996) detected an increase in dry matter production with mulching treatment. For the root economics spectrum (Ostonen et al., 2007; Weemstra et al., 2016), SRL is often regarded as a core trait reflecting the extent of soil exploration, in search of water and nutrients, per unit cost of biomass allocation (Freschet et al., 2021b). Indeed, the RL is assumed to be proportional to resource acquisition, while the biomass should be proportional to construction and maintenance (Ostonen et al., 2007; De la Riva et al., 2021). In Mediterranean plants the root economics spectrum has been identified as the main axis of variation (Montagnoli et al., 2019; De la Riva et al., 2021), but we are not aware of studies that investigated its relationship with mulching treatment.

Although SRL was higher in control area than in mulching one, we did not detect significant changes in RL and MRD (**Figures 3G, I**) at the whole 2 mm diameter class. Interestingly, when the 0.1 mm root diameter classes were considered (**Figure 5**), plants in the MU area had lower values of RL for the roots falling within the diameter range of 0.8 – 1.5 mm. This variation occurred in pioneer fine-roots representing the framework functioning in nutrient and water transport (Montagnoli et al., 2021). On the contrary, in the NM area, plants need to lengthen the fine-root system and enhance nutrient acquisition with fibrous roots, which are thin in diameter, lower in carbon costs, and higher in absorptive function overall lowering the SRL value. Moreover, we found differences in root traits at different soil depths analyzed (**Figure 4**). Mulching affected the fine-root traits with higher magnitude in the upper soil layer (0 - 10 cm). Indeed, in the upper soil layer, plants in the NM area had a lower root mass and a longer fine-root system (i.e., higher SRL) than in the MU one, although this difference was not statistically significant (**Figure 4B**). Thus, in the upper soil layer, mulching selects species with a shorter root population (i.e., lower RL) that is mostly dedicated to water transport (pioneer roots with larger diameter) and low SRL, highlighting the fundamental role played by the surface fine-roots (Baronti et al., 2022) growing with higher water and nutrient contents as in the case of the MU area (Jordán et al., 2010; Doležal et al., 2011). On the contrary, plant species of the NM area, whose soil is characterized by less water and nutrient content, have longer fine-roots and lower fine-root biomass (**Figure 4A**) since they are mostly dedicated to water and nutrient acquisition (fibrous roots). Fine-root biomass and length decreased at deeper soil layers (10 - 20, 20 - 30, and 30 - 40 cm), and the mulching effect was negligible.

According to plant species composition, mulching did not favor the development of species known as effective for PCBs phytoremediation. However, since the below-ground biomass was higher in the MU area, we could speculate that mulching facilitates the PCB phytoremediation through the increase of root system biomass development, representing the supporting backbone for the microbial communities, which in turn carry out the phytodegradation activity (Passatore et al., 2014; Terzaghi et al., 2022). Indeed, roots of some species can grow with immobile soil contaminants (PCBs, PAHs) and release cometabolites (i.e., flavonoids) during the entire lifespan (e.g., Leigh et al., 2002), fostering the growth and activity of degradative microbes. In turn, the presence of contaminants may induce a shift in the resident soil microbial community selecting the degradative populations (Mackova et al., 2010; Di Guardo et al., 2017).

Vergani et al. (2017), in a recent review study, identified plant species effective for PCB phytoremediation, and some of those species were part of the indicator species composing vegetation only of the NM area (**Table 2**). On the contrary, in the case of the MU area, these species were present but not among the indicator ones, thus suggesting that the biodiversity decline associated with the mulching application could negatively impact the diversity and abundance of native species important for phytoremediation. Since the mentioned review study (Vergani et al., 2017) focuses mainly on commercial species, we cannot exclude that there are equivalent wild native species with unknown phytoremediation potential (e.g., Slater et al., 2011). For example, *Festuca arundinacea*, a well-known species for phytoremediation, was not recorded in our study plots. However, this species has a high degree of physiognomic analogy with other dominant species found in the MU area, such as *Arrhenatherum elatius* or *Sorghum halepense* (**Table 2**). These analogies suggest the possible use of native species for testing their phytoremediation potential. In addition, native species with high phytoremediation potential (e.g., *Medicago sativa*; Mackova et al., 2010; Jing et al., 2018) could be enhanced in their coverage and associated with tools for organic pollutants stabilization (Denyes et al., 2013; Baronti et al., 2022). Finally, and more importantly, the mulching treatment seems to favor the development of a higher number of metal-tolerant plant species, which in turn could enhance the phytoremediation of polluted soils. Our findings thus indicate that the mulching practice can be a suitable method for the remediation of heavy metal-polluted soils, confirming comparable observations in forest soils (Kiikkilä et al., 2001) and the high potential of native plants for phytoremediation (Nouri et al., 2011; Wang et al., 2022). All together these findings led us to partially support our fourth hypothesis.

## 5 Conclusions

Our study pointed out that the abandonment of agricultural activities within the soil-polluted Site of National Interest (SIN Brescia-Caffaro) led to a marked increase in the soil organic carbon and pH. The over-imposed mulching (i.e., cutting the biomass and leaving the crushed clippings to decompose in situ) additionally induced a slight increase in soil nutrients. These new ecological conditions favored the establishment of a more productive plant community with a more conservative-resource strategy typical of stable hay meadows and dominated by *Arrhenatherum elatius* and *Sorghum halepense*.

Above- and below-ground plant traits showed a coordinated variation at the community level, highlighting an adaptative convergence between the leaf and root economics spectrum. Mulching selected species with a higher biomass development both above- and below-ground, while the plant community of the control area had a higher SLA and SRL. Plants subjected to the mulching treatment showed a reduction of the root population mainly functioning in nutrient and water transport (i.e., lower pioneer roots’ length).

Although mulching did not select native plant species beneficial for soil PCBs remediation, the observed increase of the root web (i.e., below-ground biomass) might be functional to the proliferation of microbes devoted to contaminants’ degradation. At the same time, mulching treatment selected a two-fold number of plant species known to be metal-tolerant, suggesting that it can be a suitable method for selecting relevant species for the remediation of HMs-polluted soils. However, our data suggest that the long-term mulching application could lead to a biodiversity decline with potential impacts also on the diversity of native species important for phytoremediation.

## Data availability statement

The raw data supporting the conclusions of this article will be made available by the authors, without undue reservation.

## Author contributions

MDF, AMo, and BC conceived the research project and developed the study plan. MDF, AMo, SAn, SAr, and BC were responsible for the field sampling. PN provided primary funding. MDF, AMo, and BC dealt with the methodological approach and the experimental design. MDF, AMo, and BC were responsible for the data collection and interpretation. AMo, PB, and AMi dealt with root sampling, processing, and data arrangement. MDF, AC, SAr, EL, and BC dealt with vegetation analysis. MDF and BC dealt with the data of leaf traits. MDF performed the data analysis and chart visualization, and wrote the manuscript draft. AMo dealt with the draft revision process. MDF and AMo finalized the manuscript. All authors contributed to the article and approved the submitted version.

## Funding

This study was funded by ERSAF Lombardia (Ente Regionale per i Servizi all’Agricoltura e alle Foreste).

## Acknowledgments

Authors thank Mario Ferrari for his valuable help in the floristic determination. MDF, AMo, and BC acknowledge the Department of Biotechnology and Life Science, University of Insubria, for providing the necessary support to the joint research project.

## Conflict of interest

The authors declare that the research was conducted in the absence of any commercial or financial relationships that could be construed as a potential conflict of interest.

## Publisher’s note

All claims expressed in this article are solely those of the authors and do not necessarily represent those of their affiliated organizations, or those of the publisher, the editors and the reviewers. Any product that may be evaluated in this article, or claim that may be made by its manufacturer, is not guaranteed or endorsed by the publisher.

## References

[B1] Ali H. Khan E. Sajad M. A. (2013). Phytoremediation of heavy metals–concepts and applications. Chemosphere 91, 869–881. doi: 10.1016/J.CHEMOSPHERE.2013.01.075 23466085

[B2] Al-Mufti M. M. Sydes C. L. Furness S. B. Grime J. P. Band S. R. (1977). A quantitative analysis of shoot phenology and dominance in herbaceous vegetation. J. Ecol. 65, 759–791. doi: 10.2307/2259378

[B3] Baronti S. Magno R. Maienza A. Montagnoli A. Ungaro F. Vaccari F. P. (2022). Long term effect of biochar on soil plant water relation and fine roots: Results after 10 years of vineyard experiment. Sci. Tot. Environ. 851, 158225. doi: 10.1016/J.SCITOTENV.2022.158225 35998720

[B4] Bartolucci F. Peruzzi L. Galasso G. Albano A. Alessandrini A. Ardenghi N. M. G.. (2018). An updated checklist of the vascular flora native to Italy. Plant Biosyst. 152, 179–303. doi: 10.1080/11263504.2017.1419996

[B5] Beesley L. Moreno-Jiménez E. Gomez-Eyles J. L. Harris E. Robinson B. Sizmur T. (2011). A review of biochars’ potential role in the remediation, revegetation and restoration of contaminated soils. Environ. pollut. 159, 3269–3282. doi: 10.1016/J.ENVPOL.2011.07.023 21855187

[B6] Benjamin J. G. Nielsen D. C. (2004). A method to separate plant roots from soil and analyze root surface area. Plant Soil 267, 225–234. doi: 10.1007/S11104-005-4887-3

[B7] Branzini A. Zubillaga M. S. (2012). Comparative use of soil organic and inorganic amendments in heavy metals stabilization. Appl. Environ. Soil Sci. 2012 doi: 10.1155/2012/721032

[B8] Bruelheide H. Dengler J. Purschke O. Lenoir J. Jiménez-Alfaro B. Hennekens S. M.. (2018). Global trait–environment relationships of plant communities. Nat. Ecol. Evol. 2, 1906–1917. doi: 10.1038/s41559-018-0699-8 30455437

[B9] Carmona C. P. Bueno C. G. Toussaint A. Träger S. Díaz S. Moora M.. (2021). Fine-root traits in the global spectrum of plant form and function. Nature 597, 683–687. doi: 10.1038/s41586-021-03871-y 34588667

[B10] Cerabolini B. E. L. Brusa G. Ceriani R. M. de Andreis R. Luzzaro A. Pierce S. (2010). Can CSR classification be generally applied outside Britain? Plant Ecol. 210, 253–261. doi: 10.1007/S11258-010-9753-6

[B11] Cerabolini B. E. L. Pierce S. Verginella A. Brusa G. Ceriani R. M. Armiraglio S. (2016). Why are many anthropogenic agroecosystems particularly species-rich? Plant Biosyst. 150, 550–557. doi: 10.1080/11263504.2014.987848

[B12] Chagas J. K. M. de Figueiredo C. C. Ramos M. L. G. (2022). Biochar increases soil carbon pools: Evidence from a global meta-analysis. J. Environ. Manage. 305, 114403. doi: 10.1016/J.JENVMAN.2021.114403 34991026

[B13] Collins C. Fryer M. Grosso A. (2006). Plant uptake of non-ionic organic chemicals. Environ. Sci. Technol. 40, 45–52. doi: 10.1021/ES0508166 16433331

[B14] Cornelissen G. Gustafsson Ö. Bucheli T. D. Jonker M. T. O. Koelmans A. A. van Noort P. C. M. (2005). Extensive sorption of organic compounds to black carbon, coal, and kerogen in sediments and soils: Mechanisms and consequences for distribution, bioaccumulation, and biodegradation. Environ. Sci. Technol. 39, 6881–6895. doi: 10.1021/ES050191B 16201609

[B15] Cousins I. T. Gevao B. Jones K. C. (1999). Measuring and modelling the vertical distribution of semi-volatile organic compounds in soils. I: PCB and PAH soil core data. Chemosphere 39, 2507–2518. doi: 10.1016/S0045-6535(99)00164-2

[B107] Dalle Fratte M. Bolpagni R. Brusa G. Caccianiga M. Pierce S. Zanzottera M. (2019a). Alien plant species invade by occupying similar functional spaces to native species. Flora 257, 151419. doi: 10.1016/j.flora.2019.151419

[B16] Dalle Fratte M. Brusa G. Pierce S. Zanzottera M. Cerabolini B. E. L. (2019b). Plant trait variation along environmental indicators to infer global change impacts. Flora 254, 113–121. doi: 10.1016/j.flora.2018.12.004

[B17] Dalle Fratte M. Pierce S. Zanzottera M. Cerabolini B. E. L. (2021). The association of leaf sulfur content with the leaf economics spectrum and plant adaptive strategies. Funct. Plant Biol. 48, 924–935. doi: 10.1071/FP20396 34366003

[B18] De Bello F. Carmona C. P. Dias A. T. Götzenberger L. Moretti M. Berg M. P.. (2021). Handbook of trait-based ecology: from theory to R tools. Cambridge University Press, Cambridge, England. doi: 10.1017/9781108628426

[B19] De Bello F. Lavorel S. Díaz S. Harrington R. Cornelissen J. H. C. Bardgett R. D.. (2010). Towards an assessment of multiple ecosystem processes and services *via* functional traits. Biodivers. Conserv. 19, 2873–2893. doi: 10.1007/s10531-010-9850-9

[B20] De Cáceres M. Legendre P. (2009). Associations between species and groups of sites: Indices and statistical inference. Ecol 90, 3566–3574. doi: 10.1890/08-1823.1 20120823

[B21] De la Riva E. G. Prieto I. Villar R. (2019). The leaf economic spectrum drives leaf litter decomposition in Mediterranean forests. Plant Soil 435, 353–366. doi: 10.1007/s11104-018-3883-3

[B22] De la Riva E. G. Querejeta J. I. Villar R. Pérez-Ramos I. M. Marañón T. Galán Díaz J.. (2021). The economics spectrum drives root trait strategies in Mediterranean vegetation. Front. Plant Sci. 12. doi: 10.3389/FPLS.2021.773118/FULL PMC8649719 34887894

[B23] Denyes M. J. Rutter A. Zeeb B. A. (2013). *In situ* application of activated carbon and biochar to PCB-contaminated soil and the effects of mixing regime. Environ. pollut. 182, 201–208. doi: 10.1016/J.ENVPOL.2013.07.016 23933124

[B24] Desie E. van Meerbeek K. de Wandeler H. Bruelheide H. Domisch T. Jaroszewicz B.. (2020). Positive feedback loop between earthworms, humus form and soil pH reinforces earthworm abundance in European forests. Funct. Ecol. 34, 2598–2610. doi: 10.1111/1365-2435.13668

[B25] Díaz S. Kattge J. Cornelissen J. H. C. Wright I. J. Lavorel S. Dray S.. (2016). The global spectrum of plant form and function. Nature 529, 167–171. doi: 10.1038/nature16489 26700811

[B26] Di Guardo A. Raspa G. Terzaghi E. Vergani L. Mapelli F. Borin S.. (2020). PCB Vertical and horizontal movement in agricultural soils of a highly contaminated site: Role of soil properties, cultivation history and PCB physico-chemical parameters. Sci. Tot. Environ. 747, 141477. doi: 10.1016/j.scitotenv.2020.141477 33076211

[B27] Di Guardo A. Terzaghi E. Raspa G. Borin S. Mapelli F. Chouaia B.. (2017). Differentiating current and past PCB and PCDD/F sources: The role of a large contaminated soil site in an industrialized city area. Environ. pollut. 223, 367–375. doi: 10.1016/J.ENVPOL.2017.01.033 28118998

[B28] Dinno A. (2017). Dunn.test: Dunn’s test of multiple comparisons using rank sums. r package version 1.3.5. Available at: https://cran.r-project.org/web/packages/dunn.test/

[B29] Doležal J. Mašková Z. Lepš J. Steinbachová D. de Bello F. Klimešová J.. (2011). Positive long-term effect of mulching on species and functional trait diversity in a nutrient-poor mountain meadow in central Europe. Agric. Ecosyst. Environ. 145, 10–28. doi: 10.1016/J.AGEE.2011.01.010

[B30] Dostál P. Březnová M. Kozlíčková V. Herben T. Kovář P. (2005). Ant-induced soil modification and its effect on plant below-ground biomass. Pedobiologia 49, 127–137. doi: 10.1016/J.PEDOBI.2004.09.004

[B31] Eissenstat D. M. Yanai R. D. (1997). The ecology of root lifespan. Adv. Ecol. Res. 27, 1–60. doi: 10.1016/S0065-2504(08)60005-7

[B32] Fort F. Jouany C. Cruz P. (2013). Root and leaf functional trait relations in poaceae species: Implications of differing resource-acquisition strategies. J. Plant Ecol. 6, 211–219. doi: 10.1093/jpe/rts034

[B33] Freschet G. T. Cornelissen J. H. C. van Logtestijn R. S. P. Aerts R. (2010). Evidence of the “plant economics spectrum” in a subarctic flora. J. Ecol. 98, 362–373. doi: 10.1111/j.1365-2745.2009.01615.x

[B34] Freschet G. T. Pagès L. Iversen C. M. Comas L. H. Rewald B. Roumet C.. (2021a). A starting guide to root ecology: strengthening ecological concepts and standardising root classification, sampling, processing and trait measurements. New Phytol. 232 (3), 973–1122. doi: 10.1111/nph.17572i 34608637 PMC8518129

[B35] Freschet G. T. Roumet C. Comas L. H. Weemstra M. Bengough A. G. Rewald B.. (2021b). Root traits as drivers of plant and ecosystem functioning: current understanding, pitfalls and future research needs. New Phytol. 232, 1123–1158. doi: 10.1111/NPH.17072 33159479

[B36] Gaisler J. Hejcman M. Pavlů V. (2004). Effect of different mulching and cutting regimes on the vegetation of upland meadow. Plant Soil Environ. 50, 324–331. doi: 10.17221/4039-PSE

[B37] Gaisler J. Pavlů L. Nwaogu C. Pavlů K. Hejcman M. Pavlů V. V. (2019). Long-term effects of mulching, traditional cutting and no management on plant species composition of improved upland grassland in the Czech republic. Grass Forage Sci. 74, 463–475. doi: 10.1111/gfs.12408

[B38] Gaisler J. Pavlů V. Pavlů L. Hejcman M. (2013). Long-term effects of different mulching and cutting regimes on plant species composition of festuca rubra grassland. Agric. Ecosyst. Environ. 178, 10–17. doi: 10.1016/j.agee.2013.06.010

[B39] Galasso G. Conti F. Peruzzi L. Ardenghi N. M. G. Banfi E. Celesti-Grapow L.. (2018). An updated checklist of the vascular flora alien to Italy. Plant Biosyst. (Cambridge, England: Cambridge University Press) 152, 556–592. doi: 10.1080/11263504.2018.1441197

[B40] Grime J. P. (1973). Competitive exclusion in herbaceous vegetation. Nature 242, 344–347. doi: 10.1038/242344a0

[B41] Grime J. P. (2006a). Plant strategies, vegetation processes, and ecosystem properties. 2nd ed (Chichester, West Sussex: John Wiley & Sons).

[B42] Grime J. P. (2006b). Trait convergence and trait divergence in herbaceous plant communities: Mechanisms and consequences. J. Veg. Sci. 17, 255–260. doi: 10.1111/j.1654-1103.2006.tb02444.x

[B43] Gupta J. P. Gupta G. K. (1986). Effect of tillage and mulching on soil environment and cowpea seedling growth under arid conditions. Arid. Land Res. Manag. 7, 233–240. doi: 10.1080/15324988709381141

[B44] Jiang L. Zhang D. Song M. Guan G. Sun Y. Li J.. (2022). The positive role of root decomposition on the bioremediation of organic pollutants contaminated soil: A case study using PCB-9 as a model compound. Soil Biol. Biochem. 171, 108726. doi: 10.1016/J.SOILBIO.2022.108726

[B45] Jílková V. (2008). The effect of ants on soil properties and processes (Hymenoptera: Formicidae related papers. Myrmecol. News 11, 191–199.

[B46] Jing R. Fusi S. Kjellerup B. v. (2018). Remediation of polychlorinated biphenyls (PCBs) in contaminated soils and sediment: State of knowledge and perspectives. Front. Environ. Sci. 6. doi: 10.3389/FENVS.2018.00079/BIBTEX

[B47] Jordán A. Zavala L. M. Gil J. (2010). Effects of mulching on soil physical properties and runoff under semi-arid conditions in southern Spain. Catena 81, 77–85. doi: 10.1016/J.CATENA.2010.01.007

[B48] Jun F. Yu G. Quanjiu W. Malhi S. S. Yangyang L. (2014). Mulching effects on water storage in soil and its depletion by alfalfa in the loess plateau of northwestern China. Agric. Water Manag. 138, 10–16. doi: 10.1016/J.AGWAT.2014.02.018

[B49] Kahmen S. Poschlod P. (2008). Effects of grassland management on plant functional trait composition. Agric. Ecosyst. Environ. 128, 137–145. doi: 10.1016/j.agee.2008.05.016

[B50] Kahmen S. Poschlod P. Schreiber K. F. (2002). Conservation management of calcareous grasslands. changes in plant species composition and response of functional traits during 25 years. Biol. Conserv. 104, 319–328. doi: 10.1016/S0006-3207(01)00197-5

[B51] Kattge J. Bönisch G. Díaz S. Lavorel S. Prentice I. C. Leadley P.. (2020). TRY plant trait database – enhanced coverage and open access. Glob. Chang. Biol. 26, 119–188. doi: 10.1111/gcb.14904 31891233

[B52] Kidd J. Manning P. Simkin J. Peacock S. Stockdale E. (2017). Impacts of 120 years of fertilizer addition on a temperate grassland ecosystem. PLos One 12, e017463. doi: 10.1371/JOURNAL.PONE.0174632 PMC5369769 28350853

[B53] Kiikkilä O. Perkiömäki J. Barnette M. Derome J. Pennanen T. Tulisalo E.. (2001). *In situ* bioremediation through mulching of soil polluted by a copper-nickel smelter. J. Environ. Qual. 30, 1134–1143. doi: 10.2134/JEQ2001.3041134X 11476489

[B54] Kurganova I. Merino A. Lopes de Gerenyu V. Barros N. Kalinina O. Giani L.. (2019). Mechanisms of carbon sequestration and stabilization by restoration of arable soils after abandonment: A chronosequence study on phaeozems and chernozems. Geoderma 354, 113882. doi: 10.1016/J.GEODERMA.2019.113882

[B55] Landolt E. Bäumler B. Erhardt A. Hegg O. Klötzli F. Lämmler W.. (2010). Flora indicativa. Ecological indicator values and biological attributes of the flora of Switzerland and the Alps. ökologische zeigerwerte und biologische kennzeichen zur flora der schweiz und der alpen. Haupt Verlag, Bern.

[B56] Lavorel S. Grigulis K. Lamarque P. Colace M. P. Garden D. Girel J.. (2011). Using plant functional traits to understand the landscape distribution of multiple ecosystem services. J. Ecol. 99, 135–147. doi: 10.1111/j.1365-2745.2010.01753.x

[B57] Leigh M. B. Fletcher J. S. Fu X. Schmitz F. J. (2002). Root turnover: An important source of microbial substrates in rhizosphere remediation of recalcitrant contaminants. Environ. Sci. Technol. 36, 1579–1583. doi: 10.1021/ES015702I/ASSET/IMAGES/LARGE/ES015702IF00004.JPEG 11999069

[B58] Leuschner C. Gebel S. Rose L. (2013). Root trait responses of six temperate grassland species to intensive mowing and NPK fertilisation: A field study in a temperate grassland. Plant Soil 373, 687–698. doi: 10.1007/S11104-013-1836-4

[B59] Leveque T. Capowiez Y. Schreck E. Xiong T. Foucault Y. Dumat C. (2014). Earthworm bioturbation influences the phytoavailability of metals released by particles in cultivated soils. Environ. pollut. 191, 199–206. doi: 10.1016/J.ENVPOL.2014.04.005 24858803

[B60] Li S. Wang F. Chen M. Liu Z. Zhou L. Deng J.. (2020). Mowing alters nitrogen effects on the community-level plant stoichiometry through shifting plant functional groups in a semi-arid grassland. Environ. Res. Lett. 15, 074031. doi: 10.1088/1748-9326/AB8A87

[B61] Macek T. Francová K. Kochánková L. Lovecká P. Ryslavá E. Rezek J.. (2004). Phytoremediation: Biological cleaning of a polluted environment. Rev. Environ. Health 19, 63–82. doi: 10.1515/REVEH.2004.19.1.63/MACHINEREADABLECITATION/RIS 15186040

[B62] Mackova M. Dowling D. Macek T. (2010). Phytoremediation rhizoremediation (Dordrecht, The Netherlands: Springer).

[B63] Mackova M. Prouzova P. Stursa P. Ryslava E. Uhlik O. Beranova K.. (2009). Phyto/rhizoremediation studies using long-term PCB-contaminated soil. Environ. Sci. pollut. Res. 16, 817–829. doi: 10.1007/S11356-009-0240-3/TABLES/7 19823887

[B64] Mašková Z. Doležal J. Květ J. Zemek F. (2009). Long-term functioning of a species-rich mountain meadow under different management regimes. Agric. Ecosyst. Environ. 132, 192–202. doi: 10.1016/j.agee.2009.04.002

[B65] McCutcheon S. Schnoor J. L. (2003). Phytoremediation: Transformation and control of contaminants (Hoboken, New Jersey: John Wiley & Sons, Inc).

[B66] McNear D. H. (2013). The rhizosphere - roots, soil and everything in between. Nat. Educ. Knowledge 4, 1.

[B67] Metsoja J. A. Neuenkamp L. Pihu S. Vellak K. Kalwij J. M. Zobel M. (2012). Restoration of flooded meadows in Estonia - vegetation changes and management indicators. Appl. Veg. Sci. 15, 231–244. doi: 10.1111/J.1654-109X.2011.01171.X

[B68] Montagnoli A. Baronti S. Alberto D. Chiatante D. Scippa G. S. Terzaghi M. (2021). Pioneer and fibrous root seasonal dynamics of vitis vinifera l. are affected by biochar application to a low fertility soil: A rhizobox approach. Sci. Tot. Environ. 751, 141455. doi: 10.1016/J.SCITOTENV.2020.141455 32889452

[B69] Montagnoli A. Dumroese R. K. Terzaghi M. Onelli E. Scippa G. S. Chiatante D. (2019). Seasonality of fine root dynamics and activity of root and shoot vascular cambium in a quercus ilex l. forest (Italy). For. Ecol. Manage. 431, 26–34. doi: 10.1016/J.FORECO.2018.06.044

[B70] Moog D. Poschlod P. Kahmen S. Schreiber K. F. (2002). Comparison of species composition between different grassland management treatments after 25 years. Appl. Veg. Sci. 5, 99–106. doi: 10.1111/J.1654-109X.2002.TB00539.X

[B71] Nadal-Romero E. Rubio P. Kremyda V. Absalah S. Cammeraat E. Jansen B.. (2021). Effects of agricultural land abandonment on soil organic carbon stocks and composition of soil organic matter in the central Spanish pyrenees. Catena 205, 105441. doi: 10.1016/J.CATENA.2021.105441

[B72] Nouri J. Lorestani B. Yousefi N. Khorasani N. Hasani A. H. Seif F.. (2011). Phytoremediation potential of native plants grown in the vicinity of ahangaran lead-zinc mine (Hamedan, Iran). Environ. Earth Sci. 62, 639–644. doi: 10.1007/S12665-010-0553-Z

[B73] Novara A. la Mantia T. Rühl J. Badalucco L. Kuzyakov Y. Gristina L.. (2014). Dynamics of soil organic carbon pools after agricultural abandonment. Geoderma 235–236, 191–198. doi: 10.1016/J.GEODERMA.2014.07.015

[B74] Oelmann Y. Brauckmann H. J. Schreiber K. F. Broll G. (2017). 40 years of succession or mulching of abandoned grassland affect phosphorus fractions in soil. Agric. Ecosyst. Environ. 237, 66–74. doi: 10.1016/j.agee.2016.12.014

[B75] Oksanen J. Blanchet F. G. Friendly M. Kindt R. Legendre P. McGlinn D.. (2022). Vegan: Community ecology package. R package version 2.6-4. Available at: https://github.com/vegandevs/vegan.

[B76] Oomes M. J. M. Olff H. Altena H. J. (1996). Effects of vegetation management and raising the water table on nutrient dynamics and vegetation change in a wet grassland. J. Appl. Ecol. 33, 576–588. doi: 10.2307/2404986

[B77] Ostonen I. Püttsepp Ü. Biel C. Alberton O. Bakker M. R. Lõhmus K.. (2007). Specific root length as an indicator of environmental change. Plant Biosyst. 141, 426–442. doi: 10.1080/11263500701626069

[B78] Passatore L. Rossetti S. Juwarkar A. A. Massacci A. (2014). Phytoremediation and bioremediation of polychlorinated biphenyls (PCBs): State of knowledge and research perspectives. J. Hazard Mater. 278, 189–202. doi: 10.1016/J.JHAZMAT.2014.05.051 24976127

[B79] Pavlů L. Gaisler J. Hejcman M. Pavlů V. v. (2016). What is the effect of long-term mulching and traditional cutting regimes on soil and biomass chemical properties, species richness and herbage production in dactylis glomerata grassland? Agric. Ecosyst. Environ. 217, 13–21. doi: 10.1016/j.agee.2015.10.026

[B80] Pérez-Harguindeguy N. Díaz S. Garnier E. Lavorel S. Poorter H. Jaureguiberry P.. (2013). New handbook for standardised measurement of plant functional traits worldwide. Aust. J. Bot. 61, 167–234. doi: 10.1071/BT12225

[B81] Pierce S. (2014). Implications for biodiversity conservation of the lack of consensus regarding the humped-back model of species richness and biomass production. Funct. Ecol. 28, 253–257. doi: 10.1111/1365-2435.12147

[B82] Prévosto B. Kuiters L. Bernhardt-Römermann M. Dölle M. Schmidt W. Hoffmann M.. (2011). Impacts of land abandonment on vegetation: Successional pathways in European habitats. Folia Geobot. 46, 303–325. doi: 10.1007/s12224-010-9096-z

[B83] Pritchard S. G. Maier C. A. Johnsen K. H. Grabman A. J. Chalmers A. P. Burke M. K. (2010). Soil incorporation of logging residue affects fine-root and mycorrhizal root-tip dynamics of young loblolly pine clones. Tree Physiol. 30, 1299–1310. doi: 10.1093/TREEPHYS/TPQ067 20668289

[B84] Ravenek J. M. Mommer L. Visser E. J.W. van Ruijven J. van der Paauw J. W. Smit-Tiekstra A.. (2016). Linking root traits and competitive success in grassland species. Plant Soil 407, 49–53. doi: 10.1007/S11104-016-2843-Z

[B85] R Core Team (2021). R: A language and environment for statistical computing. R foundation for Statistical Computing, Vienna, Austria. Available at: https://www.R-project.org/.

[B86] Reich P. B. (2014). The world-wide “fast-slow” plant economics spectrum: A traits manifesto. J. Ecol. 102, 275–301. doi: 10.1111/1365-2745.12211

[B87] Reinhold-Hurek B. Bünger W. Burbano C. S. Sabale M. Hurek T. (2015). Roots shaping their microbiome: Global hotspots for microbial activity. Annu. Rev. Phytopathol. 53, 403–424. doi: 10.1146/ANNUREV-PHYTO-082712-102342 26243728

[B88] Römermann C. Bernhardt-Römermann M. Kleyer M. Poschlod P. (2009). Substitutes for grazing in semi-natural grasslands-do mowing or mulching represent valuable alternatives to maintain vegetation structure? J. Vegetation Sci. 20, 1086–1098. doi: 10.1111/j.1654-1103.2009.01106.x

[B89] Schwinning S. Parsons A. J. (1996). Analysis of the coexistence mechanisms for grasses and legumes in grazing systems. J. Ecol. 84, 799–813. doi: 10.2307/2960553

[B90] Shen Y. Gilbert G. S. Li W. Fang M. Lu H. Yu S. (2019). Linking aboveground traits to root traits and local environment: Implications of the plant economics spectrum. Front. Plant Sci. 10. doi: 10.3389/fpls.2019.01412 PMC6831723 31737024

[B91] Simiele M. de Zio E. Montagnoli A. Terzaghi M. Chiatante D. Scippa G. S.. (2022). Biochar and/or compost to enhance nursery-produced seedling performance: A potential tool for forest restoration programs. Forests 13, 550. doi: 10.3390/F13040550

[B92] Slater H. Gouin T. Leigh M. B. (2011). Assessing the potential for rhizoremediation of PCB contaminated soils in northern regions using native tree species. Chemosphere 84 (2), 199–206. doi: 10.1016/j.chemosphere.2011.04.058 21596420 PMC3502615

[B93] Soil Survey Staff (2014). Keys to soil taxonomy. 12th ed (Washington, DC: USDA-Natural Resources Conservation Service).

[B94] Sojneková M. Chytrý M. (2015). From arable land to species-rich semi-natural grasslands: Succession in abandoned fields in a dry region of central Europe. Ecol. Eng. 77, 373–381. doi: 10.1016/J.ECOLENG.2015.01.042

[B95] Tangahu B. V. Sheikh Abdullah S. R. Basri H. Idris M. Anuar N. Mukhlisin M. (2011). A review on heavy metals (As, Pb, and Hg) uptake by plants through phytoremediation. Int. J. Chem. Eng. 2011 doi: 10.1155/2011/939161

[B96] Tasser E. Gamper S. Walde J. Obojes N. Tappeiner U. (2021). Evidence for the importance of land use, site characteristics and vegetation composition for rooting in European Alps. Sci. Rep. 11, 1–15. doi: 10.1038/s41598-021-90652-2 34045598 PMC8159984

[B97] Terzaghi E. Raspa G. Zanardini E. Morosini C. Anelli S. Armiraglio S.. (2022). Life cycle exposure of plants considerably affects root uptake of PCBs: Role of growth strategies and dissolved/particulate organic carbon variability. J. Hazard Mater. 421, 126826. doi: 10.1016/J.JHAZMAT.2021.126826 34396963

[B98] Terzaghi E. Vergani L. Mapelli F. Borin S. Raspa G. Zanardini E.. (2019). Rhizoremediation of weathered PCBs in a heavily contaminated agricultural soil: Results of a biostimulation trial in semi field conditions. Sci. Tot. Environ. 686, 484–496. doi: 10.1016/j.scitotenv.2019.05.458 31185397

[B99] Terzaghi E. Vitale C. M. Salina G. di Guardo A. (2020). Plants radically change the mobility of PCBs in soil: Role of different species and soil conditions. J. Hazard Mater. 388, 121786. doi: 10.1016/j.jhazmat.2019.121786 31836368

[B100] Tumber-Dávila S. J. Schenk H. J. Du E. Jackson R. B. (2022). Plant sizes and shapes above and belowground and their interactions with climate. New Phytol 235, 1032–1056. doi: 10.1111/nph.18031 35150454 PMC9311740

[B101] Vergani L. Mapelli F. Zanardini E. Terzaghi E. di Guardo A. Morosini C.. (2017). Phyto-rhizoremediation of polychlorinated biphenyl contaminated soils: An outlook on plant-microbe beneficial interactions. Sci. Tot. Environ. 575, 1395–1406. doi: 10.1016/j.scitotenv.2016.09.218 27717569

[B102] Wang Q. Sun Q. Wang W. Liu X. Song L. Hou L. (2022). Effects of different native plants on soil remediation and microbial diversity in jiulong iron tailings area, Jiangxi. Forests 13, 1106. doi: 10.3390/f13071106

[B103] Weemstra M. Mommer L. Visser E. J. W. van Ruijven J. Kuyper T. W. Mohren G. M. J.. (2016). Towards a multidimensional root trait framework: a tree root review. New Phytol. 211, 1159–1169. doi: 10.1111/NPH.14003 27174359

[B104] Weigelt A. Mommer L. Andraczek K. Iversen C. M. Bergmann J. Bruelheide H.. (2021). An integrated framework of plant form and function: the belowground perspective. New Phytol. 232, 42–59. doi: 10.1111/nph.17590 34197626

[B105] Wright I. J. Reich P. B. Westoby M. Ackerly D. D. Baruch Z. Bongers F.. (2004). The worldwide leaf economics spectrum. Nature 428, 821–827. doi: 10.1038/nature02403 15103368

[B106] Zanzottera M. Dalle Fratte M. Caccianiga M. Pierce S. Cerabolini B. E. L. (2020). Community-level variation in plant functional traits and ecological strategies shapes habitat structure along succession gradients in alpine environment. Comm. Ecol. 21, 55–65. doi: 10.1007/s42974-020-00012-9

