# Supplementary material for: Mulching in lowland hay meadows drives an adaptive convergence of above- and below-ground traits reducing plasticity and improving biomass: A possible tool for enhancing phytoremediation
Source: Front Plant Sci. 2022 Nov 24;13:1062911. doi: 10.3389/fpls.2022.1062911 (PMC9746715; doi:10.3389/fpls.2022.1062911)
Supplement: Supplementary file 1 [file DataSheet_1.docx]

Supplementary Material

**Supplementary Figure 1.** Main physico-chemical soil properties in relation to soil depth layers of the three points sampled in 2014 up to 100 cm depth (see Fig. 1). Legend: CEC = cation exchange capacity, SOC = organic carbon, N_tot_ = total nitrogen, S_tot_ = total sulfur, Ca = calcium, Fe = iron, Mg = magnesium, K = potassium.


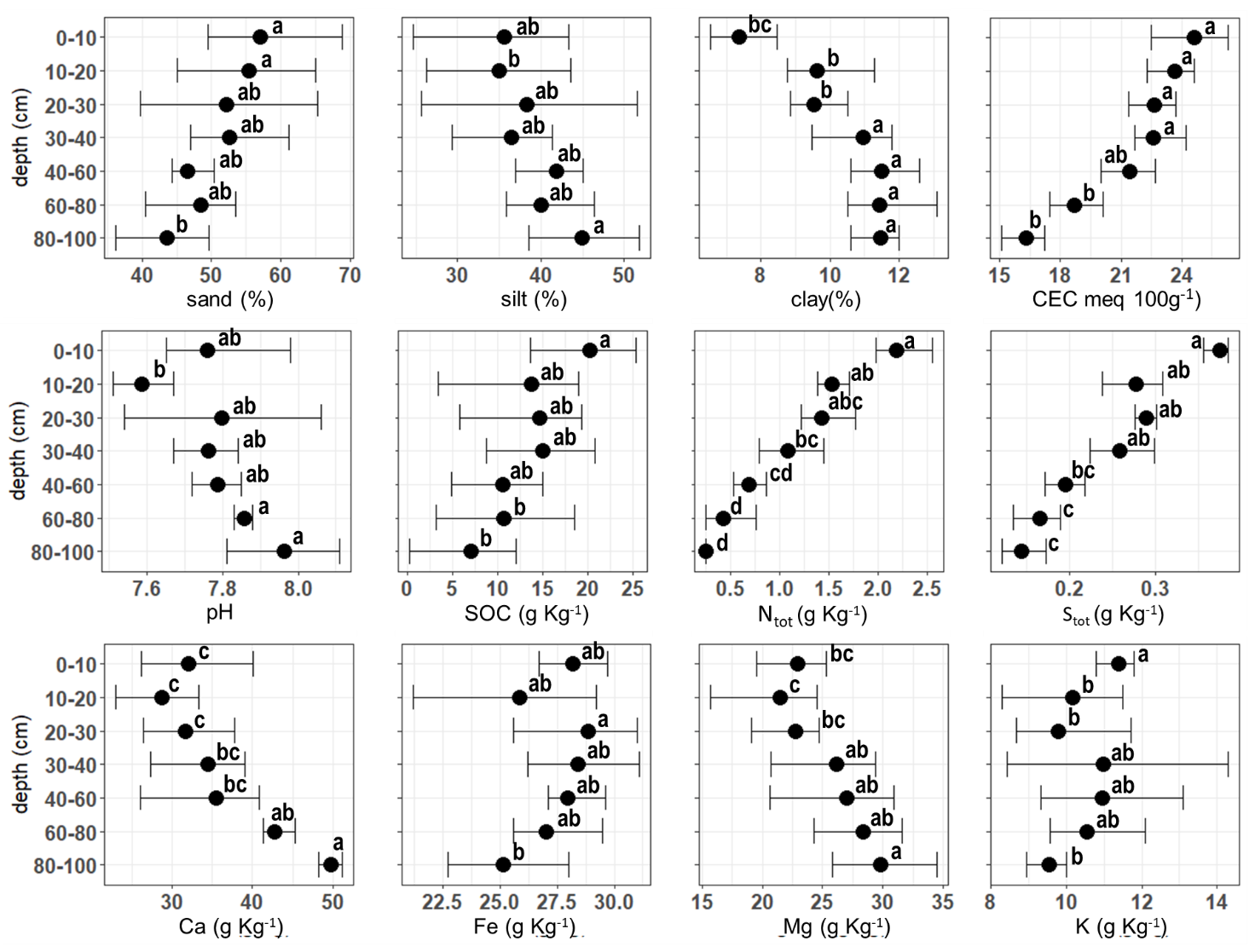


**Supplementary Table 1.** Summary statistics of the constrained ordination axes of the redundancy analysis (RDA) of the plant community composition in relation to the community-level plant functional traits that were significantly affected by the mulching treatment (see Fig. 3).

|  |  |  | **RDA1** | **RDA2** | **RDA3** | **RDA4** | **RDA5** | **RDA6** |
| --- | --- | --- | --- | --- | --- | --- | --- | --- |
| **Importance of components** | *Eigenvalue* |  | 11.41 | 4.65 | 3.82 | 3.46 | 2.20 | 2.00 |
|  | *Proportion explained* |  | 0.41 | 0.17 | 0.14 | 0.13 | 0.08 | 0.07 |
|  | *Cumulative proportion* |  | 0.41 | 0.58 | 0.72 | 0.85 | 0.93 | 1.00 |
| **Scores for constraining variables** | *Above-ground dry weight* | *AGDW* | -0.82 | -0.37 | 0.08 | -0.32 | -0.14 | -0.27 |
|  | *Below-ground dry weight* | *BGDW* | -0.67 | -0.04 | 0.17 | 0.12 | 0.71 | -0.04 |
|  | *Leaf nitrogen content* | *LNC* | 0.58 | -0.23 | -0.28 | 0.22 | -0.19 | 0.66 |
|  | *Specific leaf area* | *SLA* | 0.56 | 0.20 | -0.17 | -0.20 | -0.20 | 0.74 |
|  | *Leaf dry matter content* | *LDMC* | -0.90 | -0.32 | -0.14 | 0.16 | -0.07 | -0.17 |
|  | *Specific root length* | *SRL* | 0.82 | 0.17 | 0.22 | -0.17 | -0.47 | -0.03 |
| **Scores for species** | *Achillea roseoalba Ehrend.* | *Ach_ros* | 0.43 | 0.11 | 0.35 | 0.03 | 0.16 | -0.08 |
|  | *Arrhenatherum elatius (L.) P.Beauv. ex J.Presl & C.Presl subsp. elatius* | *Arr_ela* | -0.55 | -0.18 | -0.24 | 0.07 | -0.11 | 0.18 |
|  | *Avena barbata Pott ex Link* | *Ave_bar* | 0.55 | -0.02 | -0.34 | 0.12 | -0.07 | 0.14 |
|  | *Bellis perennis L.* | *Bel_per* | -0.05 | -0.03 | 0.48 | -0.10 | 0.29 | -0.03 |
|  | *Carex divulsa Stokes* | *Car_div* | -0.16 | 0.36 | -0.14 | 0.15 | 0.25 | -0.17 |
|  | *Cerastium brachypetalum Desp. ex Pers. subsp. brachypetalum* | *Cer_bra* | 0.26 | 0.25 | -0.09 | -0.03 | -0.25 | 0.12 |
|  | *Cirsium arvense (L.) Scop.* | *Cir_arv* | -0.42 | -0.01 | -0.29 | -0.44 | 0.06 | -0.14 |
|  | *Clematis vitalba L.* | *Cle_vit* | -0.35 | 0.26 | -0.26 | -0.19 | 0.00 | -0.03 |
|  | *Clinopodium vulgare L. subsp. vulgare* | *Cli_vul* | -0.19 | 0.63 | -0.07 | 0.10 | 0.18 | 0.02 |
|  | *Convolvulus arvensis L.* | *Con_arv* | 0.34 | 0.17 | 0.56 | -0.06 | -0.02 | 0.05 |
|  | *Convolvulus sepium L.* | *Cal_sep* | -0.56 | 0.09 | -0.10 | -0.07 | -0.09 | -0.12 |
|  | *Crepis vesicaria L.* | *Cre_ves* | 0.35 | -0.24 | -0.11 | 0.16 | 0.17 | -0.34 |
|  | *Dactylis glomerata L. subsp. glomerata* | *Dac_glo* | 0.38 | -0.09 | -0.15 | 0.37 | 0.15 | -0.05 |
|  | *Daucus carota L.* | *Dau_car* | 0.65 | 0.17 | -0.01 | -0.05 | 0.00 | 0.07 |
|  | *Erigeron annuus (L.) Desf.* | *Eri_ann* | 0.53 | 0.11 | -0.21 | 0.22 | 0.01 | -0.26 |
|  | *Galium mollugo L.* | *Gal_mol* | -0.40 | 0.00 | -0.11 | -0.13 | 0.09 | -0.15 |
|  | *Holcus lanatus L. subsp. lanatus* | *Hol_lan* | 0.21 | 0.39 | 0.34 | -0.21 | 0.00 | 0.11 |
|  | *Hypericum perfoliatum L.* | *Hyp_per* | -0.30 | 0.48 | -0.17 | 0.22 | 0.19 | 0.00 |
|  | *Lathyrus sp.* | *Lat_sp.* | -0.13 | -0.20 | 0.02 | -0.10 | 0.25 | -0.14 |
|  | *Lolium perenne L.* | *Lol_per* | 0.51 | -0.03 | -0.02 | 0.07 | 0.00 | -0.28 |
|  | *Lotus corniculatus L. subsp. corniculatus* | *Lot_cor* | 0.10 | -0.36 | -0.25 | 0.23 | 0.31 | 0.28 |
|  | *Lysimachia arvensis (L.) U.Manns & Anderb. subsp. arvensis* | *Ana_arv* | -0.03 | -0.23 | -0.06 | -0.29 | -0.01 | -0.27 |
|  | *Medicago lupulina L.* | *Med_lup* | -0.40 | 0.06 | 0.15 | 0.31 | -0.28 | -0.07 |
|  | *Medicago sativa L.* | *Med_sat* | 0.32 | 0.07 | 0.02 | 0.07 | -0.19 | -0.25 |
|  | *Myosotis arvensis (L.) Hill subsp. arvensis* | *Myo_arv* | 0.24 | 0.37 | 0.23 | -0.26 | -0.02 | 0.04 |
|  | *Picris hieracioides L. subsp. hieracioides* | *Pic_hie* | 0.13 | 0.28 | -0.25 | -0.18 | -0.16 | -0.03 |
|  | *Plantago lanceolata L.* | *Pla_lan* | 0.45 | 0.24 | 0.01 | -0.13 | -0.06 | -0.23 |
|  | *Rumex acetosa L. subsp. acetosa* | *Rum_ace* | -0.36 | -0.24 | 0.25 | 0.24 | -0.21 | -0.10 |
|  | *Rumex crispus L.* | *Rum_cri* | -0.40 | 0.30 | 0.05 | 0.38 | -0.25 | 0.00 |
|  | *Salvia pratensis L. subsp. pratensis* | *Sal_pra* | 0.51 | -0.09 | -0.14 | 0.22 | -0.19 | -0.12 |
|  | *Sorghum halepense (L.) Pers.* | *Sor_hal* | -0.61 | -0.02 | 0.01 | 0.04 | -0.01 | -0.31 |
|  | *Taraxacum F.H.Wigg. sect. Taraxacum* | *Tar_off* | -0.16 | -0.13 | -0.17 | -0.48 | -0.19 | -0.04 |
|  | *Trifolium campestre Schreb.* | *Tri_cam* | 0.13 | 0.28 | -0.25 | -0.18 | -0.16 | -0.03 |
|  | *Trifolium pratense L. subsp. pratense* | *Tri_pra* | 0.55 | -0.02 | -0.37 | 0.05 | -0.04 | 0.18 |
|  | *Trifolium repens L.* | *Tri_rep* | 0.38 | 0.03 | 0.00 | 0.01 | -0.08 | -0.24 |
|  | *Verbena officinalis L.* | *Ver_off* | -0.39 | 0.41 | -0.05 | 0.34 | -0.07 | 0.00 |
|  | *Veronica persica Poir.* | *Ver_per* | -0.33 | 0.21 | -0.11 | 0.09 | 0.33 | -0.10 |
|  | *Vicia sativa L.* | *Vic_sat* | -0.36 | -0.24 | 0.25 | 0.24 | -0.21 | -0.10 |
| ***Scores for site*** | *Mulching* | *MU1* | -1.00 | -0.87 | -0.75 | -0.26 | 2.27 | -1.14 |
|  |  | *MU2* | -1.05 | -1.81 | 1.41 | -0.23 | 0.40 | -0.60 |
|  |  | *MU3* | -1.15 | -0.74 | -1.04 | -2.64 | -0.85 | -0.19 |
|  |  | *MU4* | -1.37 | -0.37 | 1.26 | 1.69 | -2.98 | -0.02 |
|  |  | *MU5* | -1.86 | 2.82 | -1.20 | 1.55 | 1.30 | -0.57 |
|  | *No Mulching* | *NM1* | 2.44 | 0.02 | -0.34 | 0.68 | -0.82 | -3.83 |
|  |  | *NM2* | 0.96 | 1.28 | 3.36 | -1.13 | 0.98 | 1.34 |
|  |  | *NM3* | 1.41 | -1.03 | -0.95 | 1.63 | 0.88 | 0.76 |
|  |  | *NM4* | 0.66 | -0.91 | -0.78 | 0.41 | 0.15 | 3.13 |
|  |  | *NM5* | 0.97 | 1.60 | -0.97 | -1.70 | -1.33 | 1.12 |
